# Convergent somatic evolution commences in utero in a germline ribosomopathy

**DOI:** 10.1038/s41467-023-40896-5

**Published:** 2023-08-22

**Authors:** Heather E. Machado, Nina F. Øbro, Nicholas Williams, Shengjiang Tan, Ahmed Z. Boukerrou, Megan Davies, Miriam Belmonte, Emily Mitchell, E. Joanna Baxter, Nicole Mende, Anna Clay, Philip Ancliff, Jutta Köglmeier, Sally B. Killick, Austin Kulasekararaj, Stefan Meyer, Elisa Laurenti, Peter J. Campbell, David G. Kent, Jyoti Nangalia, Alan J. Warren

**Affiliations:** 1 Wellcome Sanger Institute, Wellcome Genome Campus, Hinxton, UK; 2 Wellcome MRC Cambridge Stem Cell Institute, University of Cambridge, Cambridge, UK; 3 Department of Haematology, University of Cambridge, Cambridge, UK; 4 Department of Clinical Immunology, Copenhagen University Hospital, Rigshospitalet, Copenhagen, Denmark; 5 Cambridge Institute for Medical Research, Keith Peters Building, Cambridge, UK; 6 Department of Haematology, Great Ormond Street Hospital for Children NHS Foundation Trust, London, UK; 7 University Hospitals Dorset NHS Foundation Trust, The Royal Bournemouth Hospital, Bournemouth, UK; 8 Department of Haematological Medicine, King’s College Hospital NHS Foundation Trust and King’s College London, London, UK; 9 Division of Cancer Sciences, School of Medical Sciences, Faculty of Biology, Medicine and Health, University of Manchester, Manchester Cancer Research Centre, Wilmslow Road, Manchester, UK; 10 Department of Paediatric Haematology and Oncology, Royal Manchester Children’s Hospital, Manchester Foundation Trust, Manchester, Oxford Road, Manchester, UK; 11 Teenage and Adolescent Oncology, The Christie NHS Foundation Trust, Wilmslow Road, Manchester, UK; 12 York Biomedical Research Institute, Department of Biology, University of York, York, UK

**Keywords:** Genetics research, Cancer genetics

## Abstract

Clonal tracking of cells using somatic mutations permits exploration of clonal dynamics in human disease. Here, we perform whole genome sequencing of 323 haematopoietic colonies from 10 individuals with the inherited ribosomopathy Shwachman-Diamond syndrome to reconstruct haematopoietic phylogenies. In ~30% of colonies, we identify mutually exclusive mutations in *TP53*, *EIF6*, *RPL5*, *RPL22*, *PRPF8*, plus chromosome 7 and 15 aberrations that increase *SBDS* and *EFL1* gene dosage, respectively. Target gene mutations commence in utero, resulting in a profusion of clonal expansions, with only a few haematopoietic stem cell lineages (mean 8, range 1-24) contributing ~50% of haematopoietic colonies across 8 individuals (range 4-100% clonality) by young adulthood. Rapid clonal expansion during disease transformation is associated with biallelic *TP53* mutations and increased mutation burden. Our study highlights how convergent somatic mutation of the p53-dependent nucleolar surveillance pathway offsets the deleterious effects of germline ribosomopathy but increases opportunity for *TP53*-mutated cancer evolution.

## Abstract (web summary)

Shwachman-Diamond Syndrome (SDS) is an inherited ribosome assembly disorder that increases the risk for haematopoietic malignancies. Here, the authors analysed clonal selection and evolution in SDS by sequencing patient-derived haematopoietic stem/progenitor cell colonies and exploring the function of key drivers in model organisms.

## Introduction

All cells acquire somatic mutations over time through a range of exogenous and endogenous DNA damaging processes. The tracking of such mutations has enabled the reconstruction of lineage histories of individual haematopoietic stem cells (HSC) to chart clonal dynamics in healthy and malignant human haematopoiesis over life^1–4^. These studies have shown that some HSCs gain a fitness advantage over others, typically through acquisition of certain somatic mutations, resulting in slow but continuous clonal expansion over a lifetime^3^. By the 7th to 8th decade of life, there is a collapse in HSC clonal diversity in blood with many clonal expansions driven by mutations in a range of genes (e.g. *DNMT3A*) and copy number changes (e.g. loss of Y)^3,5,6^. Relatively little, however, is understood about how clonal selection and population dynamics differ in individuals born with germline mutations that compromise haematopoiesis and confer an increased risk of blood cancer.

Shwachman-Diamond syndrome (SDS) is an inherited ribosome assembly disorder caused by compound heterozygous germline mutations in the *SBDS* gene, typically the combination of one null and one hypomorphic allele^7–9^. The wild-type SBDS protein cooperates with the GTPase EFL1 to catalyse release of the anti-association factor eIF6 from the intersubunit face of the large ribosomal subunit to promote ribosome maturation and recycling^8–12^. The resulting ribosome assembly defect and reduced protein synthesis results in bone marrow failure (BMF), with over one-third of individuals subsequently developing myelodysplasia (MDS) and acute myeloid leukaemia (AML) by the fourth decade of life^13,14^.

A number of recurrent somatic genetic events have been identified in SDS. In individuals with one null and one hypomorphic *SBDS* allele on chromosome (chr) 7q, copy number neutral loss of heterozygosity (LOH) increases the gene dose of the hypomorphic *SBDS* allele c.258 + 2T → C and replaces the null allele^15,16^. Similarly, uniparental disomy can occur on chr15 to mitigate against the more damaging compound heterozygous *EFL1* mutation combinations in SDS^17^. Chr20q deletion and *EIF6* point mutations also reduce eIF6 dosage and/or its affinity for the ribosome^14,18–21^. Each of these genetic events likely compensate for defective SBDS function in SDS by restoring ribosome homoeostasis.

Impaired ribosome assembly stabilises the tumour suppressor protein p53 via the nucleolar surveillance pathway (NSP)^22^. Increased p53 expression is observed in haematopoietic cells from individuals with SDS^23^ and targeted disruption of *Sbds* in murine models causes p53-dependent induction of apoptosis in haematopoietic progenitor cells^24,25^. Indeed, *TP53* mutations are recurrent across, and within, individuals with SDS^18,26^. Since *TP53* is the most frequently altered gene in human tumours^27^, with mutations arising both early in tumorigenesis, such as in glioblastoma and ovarian cancers^28–30^, as well as late during cancer progression^28,31^, it is critical to understand the impact of *TP53* mutations on cellular competition. SDS provides a unique window into understanding the earliest stages of *TP53*-mutated clonal selection due to the selective pressure imposed by the germline *SBDS* mutation.

In this study, we use whole-genome sequencing (WGS) of single-cell-derived haematopoietic colonies to interrogate the mutational consequences, selection landscape and clonal dynamics in the germline ribosomopathy, SDS. We show that *TP53* and the p53-dependent nucleolar stress pathway are a frequent target of mutually exclusive, convergent somatic mutation from early life, including in utero. These mutations drive early loss of clonal diversity that while offsetting the deleterious effects of defective ribosome assembly, increases the propensity for *TP53*-mutated cancer evolution.

## Results

### Premature and marked loss of haematopoietic clonal diversity in SDS

**Fig. 1:**
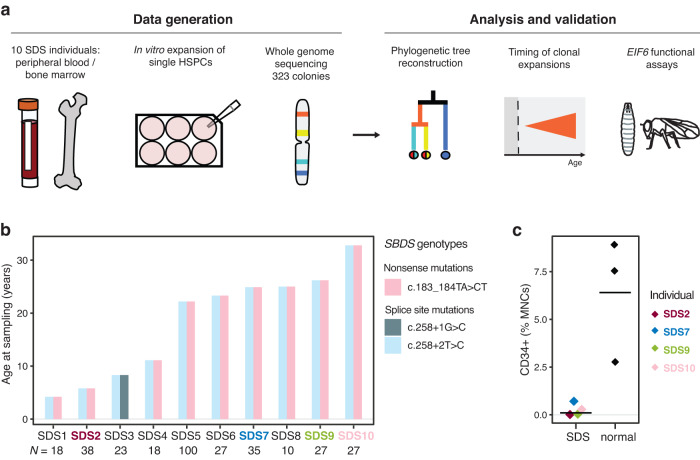
Study design and cohort. **a** Schematic of experimental design. Single haematopoietic stem and progenitor cells (HSPC) and mononuclear cells (MNCs) from peripheral blood or bone marrow from individuals with SDS were expanded into colonies in vitro and each colony underwent whole-genome sequencing (WGS). Somatic mutations were used to reconstruct haematopoietic phylogenies. The timing of acquisition, clonal dynamics and functional consequences were investigated for driver mutations associated with SDS. Inkscape. **b** Age at sampling and *SBDS* genotype for each individual with SDS. The two bi-coloured columns represent the two parental alleles, with all individuals having biallelic germline mutations in *SBDS*. Highlighted samples (SDS2, SDS7, SDS9 and SDS10) were measured for frequency of CD34+ HSPCs, shown in (**c**). *N* the number of haematopoietic colonies analysed per individual. **c** Frequency of CD34+ HSPCs in bone marrow samples (expressed as a % of total viable MNCs) was analysed by flow cytometry in four of the individuals with SDS and three healthy individuals (black). Source data are provided as a Source Data file.

We studied ten individuals with SDS aged 4–33 years, who harboured biallelic germline loss-of-function mutations in the *SBDS* gene. We isolated single haematopoietic stem and progenitor cells (HSPC) and mononuclear cells (MNCs) from peripheral blood or bone marrow from individuals, and following whole-genome sequencing of single-cell-derived colonies (*n* = 323), we used the somatic mutations to reconstruct haematopoietic phylogenies, using published methodology^4^ (Fig. 1a, b). Individuals had typical clinical features of SDS including neutropenia, pancreatic insufficiency and osteopenia, presenting early in life with failure to thrive. Histomorphology revealed bone marrow hypocellularity (range 10–40%), dyserythropoiesis, and decreased granulopoiesis with a reversed myeloid-erythroid ratio (1:3–4). One individual (SDS8) had progressed to MDS with trilineage dysplasia shortly before sampling. Flow cytometric phenotyping of bone marrow (BM) and peripheral blood (PB) mononuclear cells showed a reduced frequency of total CD34^+^ progenitors in individuals with SDS (median 0.2%) compared to healthy bone marrow donors (median 7.5%) (*t*-test *p* = 0.01; Fig. 1c and Supplementary Fig. 1), consistent with previous studies^32^. We undertook WGS of 323 individual single-cell-derived colonies seeded from haematopoietic stem and progenitor cells (HSPC), to a mean depth of 20x reads, together with matched buccal swab DNA as a germline reference, in all individuals. Somatic mutations together with embryonic variants were identified through a combination of mutation calling using a matched germline reference as well as an unmatched variant calling approach, as detailed in the methods. Haematopoietic colonies were clonally derived with a somatic mutation mean variant allele fraction (VAF) of >0.4, thus representing the somatic mutations present in the single-cell that seeded the colony. In total, we identified 118,564 single nucleotide variants, 6287 small insertions and deletions, 74 structural variants and 5 chromosomal copy number aberrations across the cohort (Supplementary Dataset 1). The number of SNVs per colony per individual ranged from a median of 130 (range 99–163) in the youngest (age 4 years) to a median of 714 (range 593–744) for one of the older individuals (age 25 years) with MDS (SDS8).

**Fig. 2:**
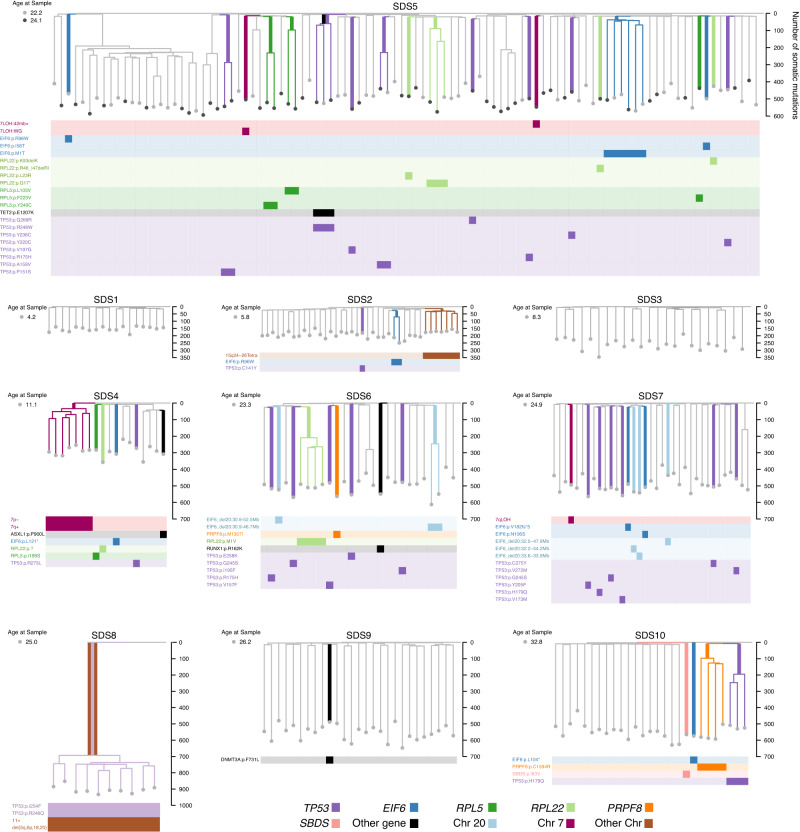
Recurrent mutations across SDS haematopoietic phylogenies. Phylogenetic trees of haematopoietic colonies for ten individuals with SDS. Each individual had between 10–100 colonies sequenced and included for phylogenetic analysis. Branches with somatic mutations in driver genes previously reported and/or under positive selection in this study are coloured. Branches with known driver mutations of clonal haematopoiesis are shown in black, and those associated with SDS in other colours. The branch harbouring the driver mutation is shown with a thicker coloured line. The *Y*-axis shows the total number of somatic mutations including driver mutations. Rows beneath phylogenetic trees show the specific driver mutations, with colonies harbouring that mutation more densely coloured. Of note, no known driver mutations were detected amongst the somatic mutations found in SDS1 and SDS3. *SDS8 was diagnosed with transformation to myelodysplasia with trilineage dysplasia 1 month before sampling. All SDS8 colonies had a complex karyotype with many chromosomal (Chr) copy number aberrations (CNA). CNAs confidently shared across SDS8 colonies are shown on the tree.

Somatic mutations from individual colonies were used to reconstruct phylogenetic trees of haematopoiesis (Fig. 2). We identified a profusion of clonal expansions in 7 of 10 individuals (SDS2, SDS4, SDS5, SDS6, SDS7, SDS8 and SDS10), an observation highly uncharacteristic of healthy haematopoiesis in individuals <70 years of age or individuals with blood cancers studied to date^1,3,4^. Given that by birth, blood cells typically have already acquired around 50–65 somatic mutations^3^, we defined post embryonic clonal expansions as any clade comprising ≥2 colonies, whose common ancestor was observed after 75 mutations from the start of the phylogenetic trees. We identified 18 such clonal expansions of varying sizes across the trees, representing 21% of colonies (Supplementary Fig. 2 and Supplementary Dataset 2).

### Somatic mutations under selection in individuals with SDS

The congenital ribosomopathy SDS provides strong selective pressure for the expansion of HSCs that have accumulated fitness-enhancing somatic mutations^14,15,17–21,26^. We observed several genomic events that directly target *SBDS*, identifying four instances of chr7q LOH, each resulting in an extra copy of the c.258 + 2T > C donor splice site mutant hypomorphic *SBDS* allele (SDS5, 4, 7, Fig. 2) and one somatically acquired nonsynonymous *SBDS* mutation, also occurring on the hypomorphic allele (SDS10, Fig. 2). We also identified a chr15 event (15q24-26 tetra) that doubles the copy number of the *EFL1* gene located at 15q25.2. These somatic events appear to be directly compensating for the germline defect that impairs the cooperation between SBDS and EFL1 that is required for ribosome maturation^8^.

**Fig. 3:**
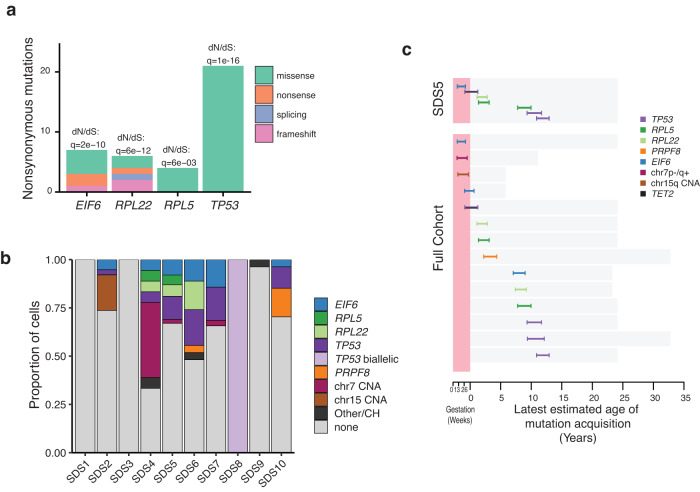
Detecting positive selection and timing of mutation acquisition. **a** Number of nonsynonymous mutations in the four genes under significant positive selection (ratio of normalised nonsynonymous mutations (dN) to normalised synonymous mutations (dS) dN:dS > 1, *q* < 0.01). **b** Proportion of cells per individual carrying driver mutations classified by gene and chromosomal abnormality. CH genes associated with clonal haematopoiesis, CNA copy number alteration. **c** Timing of division of the most recent common ancestor (MRCA) of clonal expansions (clade comprising 2 or more colonies) harbouring driver mutations. This gives the latest time point (together with 95% credibility interval) by which the driver mutation was acquired as represented by the inferred timing of the end of the shared branch, but it is possible that the driver mutation occurred at any time along the branch that harbours the driver mutation. The top panel illustrates the diversity of timings within a single individual (SDS5) and the bottom panel shows the timing of all the identified driver based expansions in the full cohort (including SDS5) with the exception of SDS8. A total of 14 branches harbouring driver mutations from the cohort are timed for their latest age of acquisition. The bars span the 95% credibility interval of the MRCAs. Source data are provided as a Source Data file.

More commonly, we observed frequent and independently acquired somatic mutations affecting five other genes. Three of these genes have been reported as recurrently mutated in SDS (*PRPF8, TP53, EIF6*)^18,21,26^. In addition, we identified somatic nonsynonymous mutations under positive selection in the *RPL5* and *RPL22* genes (ratio of normalised nonsynonymous (dN) to normalised synonymous mutations (dS) dN:dS >1, *q* < 0.01) (Figs. 2 and 3a), both encoding protein components of the large ribosomal subunit. Overall, we identified 24 independent missense mutations in *TP53* (Supplementary Fig. 3), by far the most commonly mutated gene in the cohort, and 1 start codon loss, 4 missense, 2 nonsense, 1 frameshift mutation and 5 gene deletions in *EIF6*. The somatic mutations in *RPL22* suggested loss of function (1 start codon loss, 1 nonsense, 2 splice site, 1 missense and 2 in frame deletions), while *RPL5* and *PRPF8* mutations were missense SNVs (*n* = 4 and 2 respectively). Mutations in *TP53*, *EIF6*, *RPL5* and *RPL22* genes were recurrent within the same individual, with 9 different *TP53* mutations observed in SDS5 and 5 independent *EIF6* mutations occurring in SDS7 (Fig. 2). In addition to recurrent mutations in *PRPF8*, *TP53*, *EIF6*, *RPL5* and *RPL22*, we observed mutations in *DNMT3A, ASXL1, TET2* and *RUNX1* associated with clonal haematopoiesis (CH), consistent with the study by Kennedy et al.^18^. We term recurrent mutations in either SDS-associated or known genes of CH^33,34^/haematological cancers^35^ as driver mutations (see “Methods”). Across all the individuals in this study who had a mean age of only 18 years (4–33 years range), 31% of colonies (101/323) harboured a driver mutation (Fig. 2 and Supplementary Fig. 2).

There was heterogeneity in both the frequency of driver mutations and the degree of clonal expansion across individuals. No driver mutations were identified in 67 of 68 haematopoietic colonies from three individuals (SDS1, SDS3 and SDS9). In contrast, 46% of the remaining 255 colonies from 7 individuals harboured driver mutations (median 48%, range: 26–100%; Figs. 2 and 3b). The three individuals with a sparsity of driver mutations also lacked detectable clonal expansions (SDS1, SDS3 and SDS9). We did not observe any correlation between the prevalence of clonal expansions or driver mutations and peripheral blood count cytopenias (Supplementary Table 1).

We explored clonal expansions lacking driver mutations as well as non-expanded branches for somatic mutations in additional putative target genes. We observed a large embryonic clonal expansion in SDS5 comprising 21 colonies which harboured a chromosomal translocation affecting *GPR137B*, coding for an mTORC1 regulatory protein. Nonsynonymous somatic variants were also identified in genes involved in translation (*EIF4A1* and *EIF5AL1*), RNA metabolism (*DDX23*, *DDX42*, *DDX60*, and *DDX39B*), and ribosomal proteins (*RPS14* and *RPS21*) (Supplementary Dataset 1). Since these mutations were only observed in single colonies, their potential pathogenicity remains unclear.

Apart from one individual (SDS8) with clonal evolution to biallelic *TP53* mutations and MDS transformation, and one individual (SDS5) with a concurrent *TET2* mutation within a *TP53*-mutated clade, we did not observe any instances where more than one driver mutation was present within the same lineage (Fig. 2). Colonies harbouring copy number alterations that compensated for SBDS or EFL1 dosage (SDS2, SDS4, SDS5 and SDS7) were also mutually exclusive with colonies harbouring nucleotide substitutions in driver genes. This suggests that a single heterozygous mutation in one of several target genes is sufficient to provide a fitness advantage in the context of the germline ribosome assembly defect.

In total, 131 of 323 colonies (41%) were either in an expanded lineage and/or harboured a driver mutation (median across individuals 37%, range 0–100%; Supplementary Fig. 2). Excluding the 2 young individuals without clonal expansions or driver mutations (SDS1 and 3), a median of ~50% of haematopoietic colonies in individuals harboured a driver mutation or were part of expanded lineages (range 4–100%). Assuming that single colonies with driver mutations also represent small clonal expansions, on average, 8 expanded HSC lineages (range 1–24 HSC lineage expansions across 8 individuals) were producing half of the haematopoietic cells sampled in these individuals. The oligoclonality in young individuals with SDS is in stark contrast to healthy/non-SDS haematopoiesis, where such a degree of clonal expansion is not observed until after the age of 70 years^3^.

### Timing haematopoietic clonal expansions in individuals with SDS

Due to the linear acquisition of somatic mutations over time, we can use the phylogenetic trees to estimate when clonal expansions driven by driver mutations commenced in life. This is possible by converting the number of somatic mutations acquired by the most recent common ancestor of a clonal expansion that shares a driver mutation to chronological age (see methods). We timed the start of 14 different clonal expansions driven by mutations in *TP53, RPL5, RPL22, PRPF8, EIF6*, as well as copy number events affecting *SBDS* and *EIF6* (Fig. 3c). They exhibit a range of clonal expansion times over life, commencing from early in utero up to age 12. With the exception of the copy number events affecting *SBDS* and *EFL1* on chromosomes 7 and 15, both of which appear to have occurred in utero with very early clonal expansions, the timing of expansions was independent of the different targets such as *TP53*, *RPL5*, *RPL22, PRPF8* and *EIF6* (Fig. 3c). We note however, that our study is biased towards detecting earlier somatic driver mutation acquisitions due to the longer duration for clonal expansion. It is therefore plausible that somatic driver mutations and clonal expansions may continue beyond childhood but are not captured by our sampling.

### Biallelic *TP53* mutation and transformation to myelodysplasia

**Fig. 4:**
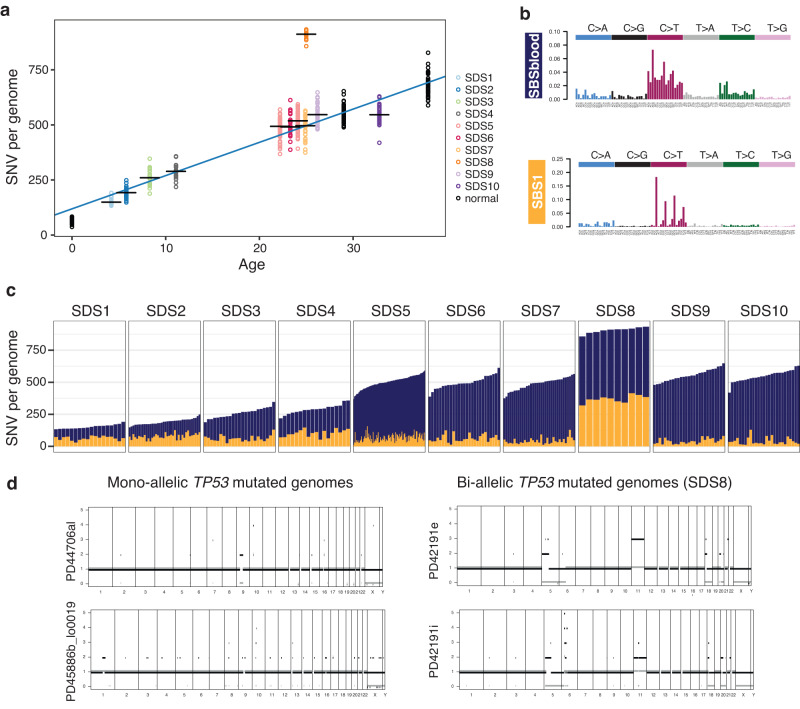
Mutation burden, mutational processes and biallelic *TP53* variants. **a** Mutation burden (number of SNVs) as a function of age for ten individuals with SDS. Each circle represents one colony genome, with the black horizontal bars representing the median burden per individual. Two timepoints from SDS5 are shown at different ages. Circles coloured black (normal) representing mutation burdens from three haematopoietically healthy (non-SDS) individuals (published data^3^) are shown for comparison. The blue line represents the regression line through the colonies from individuals with SDS. **b** Trinucleotide context of somatic mutations. The two mutational signatures were identified across all genomes. SBS1^42, 43^ is characterised by spontaneous deamination of cytosines, and the second mutational signature, termed SBSBlood^1, 2, 41^ represents mutations typical of endogenous mutations in HSCs. **c** Number of SNVs attributable to the mutational signatures SBS1 (green) and SBSblood (blue) across each colony from each individual with SDS. Each bar represents the genome from one colony. Note SDS8 has a higher total mutation burden due to increased SBS1 mutations. **d** Copy number variation for two representative colony genomes with heterozygous/mono-allelic *TP53* mutation (from different individuals) and two clonally related colonies from SDS8 with biallelic *TP53* mutations. Ploidy is shown on the *y*-axis and genomic location on the *x*-axis, for the two parental alleles (green and red). CNA copy number aberration. Source data are provided as a Source Data file.

Within the cohort, one individual (SDS8) was diagnosed with MDS at age 25 years following the development of pancytopenia with morphological trilineage dysplasia and increased bone marrow reticulin. Molecular karyotyping revealed biallelic *TP53* mutations with a complex karyotype. The phylogenetic tree from this individual, reconstructed from blood <1 month post diagnosis of MDS, confirmed a large clonal expansion that dominated the myeloid haematopoietic compartment with individual colonies harbouring concurrent *TP53* I254F and R248Q mutations. Biallelic *TP53*-mutated colonies also harboured a profusion of CNAs including chr5-, 6p-, 11+, 18-, 20-, and X- (Figs. 2 and 4d), many of which were also confirmed by clinical karyotyping. These CNAs were not observed in other individuals or genotypes, including heterozygous/mono-allelic *TP53*-mutated colonies (Fig. 4d), but are a well-recognised feature of transformation to MDS/AML in SDS^36^ and are consistent with the CNAs that are recognised to be associated with biallelic *TP53* mutant clones in cancer more generally^37,38^.

Mutation burden in SDS8 colonies was significantly higher than that expected for the age of this individual. Excluding SDS8, SNV accumulation in SDS in colonies, both with and without driver mutations, was ~15 substitutions per year (CI_95%_ = 13–17, linear mixed effects model, *p* = 1 × 10^−13^, Fig. 4a), comparable to that reported for healthy haematopoiesis^1–3,39–41^. In contrast, SNV burden in SDS8 colonies was more than double that expected for age (*p* = 6.867e−13, mean = 905, range = 857–933, compared to an expected 496, Fig. 4a, c). To assess what mutational processes might be driving this increase in mutational burden in SDS8, we inferred mutational signatures across the cohort. Similar to healthy/non-SDS blood, we identified two mutational signatures: SBS1^42,43^, characterised by spontaneous deamination of cytosines; and “SBSBlood” which identifies typical endogenous mutations in HSCs^1,2,41^ (Fig. 4b). Biallelic *TP53***-**mutated/CNA genomes from SDS8 had a substantially higher proportion and total burden of SBS1 mutations (mean = 41%, range = 37–45%) than expected for age (expected = 12%; Fig. 4c). As SBS1 mutations are known to occur during cell division, this increase in SBS1 mutations in SDS8 suggests very rapid clonal expansion compared to other SDS individuals.

We next explored when the transition to rapid clonal expansion may have commenced in SDS8 relative to clinical presentation with MDS. The shorter end branches of the clonal expansion in SDS8 had a distinct mutational profile (Supplementary Fig. 4a), characterised by a marked increase in SBS1 (65% of mutations in end branches, Supplementary Fig. 4b). The shared trunk of the SDS8 phylogeny was more similar to the mutational spectrum of other individuals of a similar age in the study, albeit with a mild increase in SBS1 (27% of shared mutations) (Supplementary Fig. 4a, b). This raised the possibility that the transition from normal mutation acquisition or cell division rates in SDS8 to rapid clonal expansion occurred at some point along the shared trunk of the SDS8 phylogeny. In order to estimate when along this shared branch such a transition may have occurred, we decomposed the mutational spectrum of the shared branch into the sum of two mutational profiles—the mutation profile of SDS haematopoiesis observed in other SDS individuals of a similar age (composite age-matched SDS signature) and the mutational spectrum in the private end branches of SDS8 (transformation signature) as described in “Methods”. The combination of these two profiles accurately reconstructed the mutation spectrum of the shared trunk (composite age-matched SDS signature 0.80, transformation signature 0.20, cosine similarity 0.958, methods, Supplementary Fig. 4c). This provides a rough estimate for when rapid growth may have commenced, assuming this occurred at a single time point historically and suggested a very recent age of transformation of 23.5 years (95% CI 19.2–24.9). Even the lower bound of this age range implies rapid clonal outgrowth. Assuming a very simple model of a single clone expanding at a constant rate to clonal dominance (see “Methods”), it would suggest that this clone was growing by 5200% (150%–15,000%) per year, corresponding to the mutant HSC clone size doubling roughly every 2 months. These data highlight the potential rapidity of transformation to MDS in this individual with SDS and provide a potential explanation for the often abrupt disease progression that may be observed clinically. However, it is important to note that we have only characterised transformation to MDS in a single individual. Analysis of further individuals is required to confidently estimate the trajectory to disease transformation in SDS. Of interest, we found no evidence that heterozygous *TP53-*mutated colonies across the cohort increased mutation burden (linear mixed model *p* = 0.22, all comparisons with *TP53*: Tukey *p* ≥ 0.87) suggesting that the rapid clonal expansion and copy number aberrations observed in SDS8 were driven by biallelic mutation of *TP53* and/or the chromosomal aberrations present.

### Functional impact of *EIF6* mutations

Mutations in the *EIF6* gene confer a fitness advantage to SBDS-deficient cells^8,18,21,44^. Of the 13 *EIF6* mutational events identified, start-codon loss (M1T), missense mutations (I58T, R96W, N106S), nonsense mutations (L121*, L104*), frameshift truncating mutations (V182fs*5), and deletions were observed. While R96W and M1T were associated with clonal expansions, the remaining events affected single colonies. The differences both in somatic variants and clone size suggest variable functional effects of individual *EIF6* mutations.

**Fig. 5:**
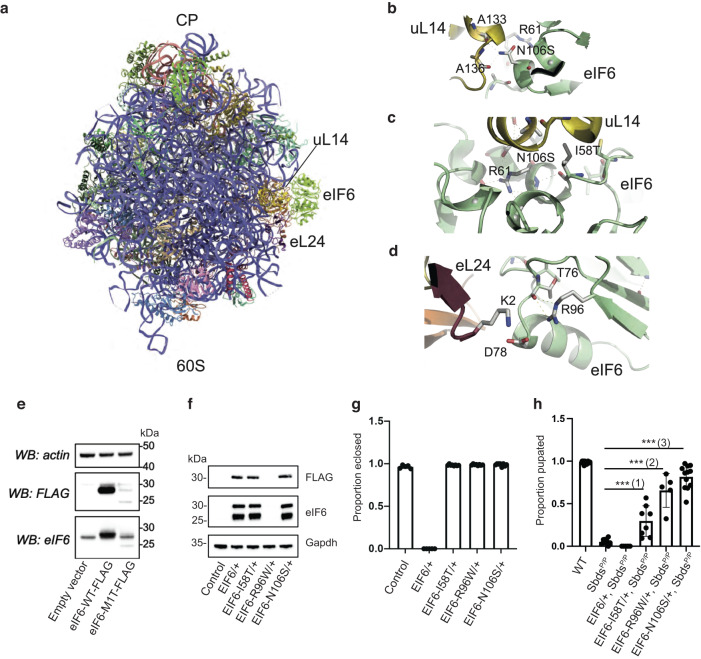
Functional consequences of *EIF6* variants. **a** Atomic model of human eIF6 bound to the 60S ribosomal subunit (PDBID: 7OW7). CP central protuberance. Stabilising interactions formed by eIF6 residues N106 (**b**), I58 (**c**) and R96 (**d**) are predicted to be lost with somatic mutation. Figures were generated using Pymol v1.2. eL24 is coloured salmon; uL14, gold; eIF6, green. **e** Cell extracts from HEK293T cells expressing empty vector, human eIF6-WT-FLAG or eIF6-M1T-FLAG mutant for 24 h were immunoblotted to detect eIF6, FLAG and actin as loading control. **f**, **g** Overexpression of eIF6 variants in WT flies. Genotypes of fly samples are indicated in Supplementary Table 2. **f** Extracts from larvae with the stated genotypes were immunoblotted to detect the indicated proteins (minimum 3 replicates). Control, da-GAL4 line. **g** Proportion of indicated fly genotypes that eclosed (minimum 5 replicates, minimum *n* = 216; error bars represent mean ± SD). **h** Genetic complementation of Sbds-deficient *Drosophila* (*Sbds* ^*P/P*^) with SDS-related eIF6 variants. Proportion of indicated genotypes developing to the pupal stage is shown (minimum 5 replicates, minimum *n* = 256; error bars represent mean ± SD; ***two tailed *t*-test, *p*(1) = 0.00060711; *p*(2) = 2.56426E−07; *p*(3) = 2.3141E−13. Source data are provided as a Source Data file.

To study the functional consequences of eIF6 mutations on ribosome assembly, we mapped residues I58, N106 and R96 to the 2.4 Å cryo-EM structure of the human eIF6-60S complex (PDBID: 7OW7) (Fig. 5a–d). The eIF6 residue N106 lies at the interface between eIF6 and the 60S ribosomal subunit protein uL14, forming hydrogen (H)-bonding interactions with the backbone oxygen atoms of uL14 residues A133 and A136 (Fig. 5b). N106S likely reduces the hydrogen bonding interface between eIF6 and uL14 to aid its dissociation^21^. Similarly, the side chain of residue I58 forms hydrophobic interactions with uL14, while the main chain oxygen of I58 forms an intra-protein H-bond with the main chain nitrogen of R61 which in turn forms a series of intra-protein H-bonds with the side chain and backbone atoms of N106 (Fig. 5c). Replacement of isoleucine with the more polar threonine side chain in the I58T variant may increase solvation and reduce the stability of the eIF6-uL14-interface. Indeed, eIF6 variants I58T and N106S reduce the affinity of eIF6 for the 60S subunit across yeast, *Dictyostelium* and human cells^8,21^. Similarly, the side chain of R96 stabilises the polar interaction between eIF6 (residue D78) and ribosomal protein eL24 (residue K2) which is likely lost by the replacement of arginine with tryptophan in the R96W variant (Fig. 5d).

To assess expression of the eIF6 M1T variant, we performed immunoblotting of extracts from human HEK293T cells expressing wild-type (WT) or FLAG-tagged eIF6-M1T protein. We confirmed that the start codon loss variant eIF6-M1T significantly reduced eIF6 expression as anticipated (Fig. 5e), further indicating that a subset of *EIF6* missense variants reduces the dose of eIF6^18,21^. Nonsense (L104*, L121*) variants, deletion-causing frameshift mutations, or genomic eIF6 deletions, would also be expected to reduce eIF6 dosage due to *EIF6* haploinsufficiency. Immunoblotting of *Drosophila* larval cell extracts revealed that expression of the eIF6-I58T and eIF6-N106S mutants was comparable to eIF6-WT, but expression of the eIF6-R96W variant was reduced (as detected by anti-FLAG antibody) (Fig. 5f). Total eIF6 expression (FLAG-tagged variant plus endogenous eIF6 protein), as detected by anti-eIF6 antiserum, was comparable for eIF6-WT, eIF6-I58T or eIF6-N106S variants, but undetectable for eIF6-R96W (Fig. 5f below). These data indicate that transgenic overexpression of eIF6 WT or variants does not significantly induce expression of the endogenous eIF6 protein. Importantly, overexpression of WT eIF6 but not the eIF6 variants (N106S, R96W and I58T), reduces the viability of WT flies (Fig. 5g), further demonstrating that overexpression of eIF6 variants does not result in functionally significant induction of the endogenous *Drosophila* eIF6 protein.

We next tested the ability of the eIF6-I58T, eIF6-R96W and eIF6-N106S mutants to rescue the larval lethality of SBDS-deficient (*Sbds* ^*P/P*^) *Drosophila*^21^ compared to WT eIF6. Homozygous Sbds-deficient flies exhibited a severe growth defect, with only 5% of larvae surviving to the early pupal stage (Fig. 5h). While wild-type eIF6 failed to rescue the lethal Sbds-deficient phenotype, eIF6-R96W, eIF6-N106S, and to a lesser extent, eIF6-I58T, rescued a proportion of flies that survived to the late pupal stage (Fig. 5h). Taken together with previous genetic experiments in yeast^8^, our data suggest that different eIF6 variants have significant but variable cellular rescue potency in SDS. We conclude that somatic *EIF6* mutations compensate for the ribosome maturation defect in SDS either by reducing the *EIF6* gene copy number present in the cell, or by modulating the level of eIF6 protein or its 60S subunit binding function, resulting in at least partial restoration of ribosome homoeostasis in SDS.

## Discussion

In this study, we have shown that the strong selective pressure to overcome impaired ribosome biogenesis and avoid p53-mediated cell death in SDS results in convergent somatic mutations in a unique set of target genes, not observed in the context of ageing^45–47^ or haematological perturbations such as autoimmunity^48^ or chemotherapy^49^. The survival advantage conferred by such mutations results in HSC clonal expansions, often commencing very early in life, even in utero, in individuals with SDS. By young adulthood and even childhood, we estimate that nearly half of haematopoiesis is derived from a small number of expanded HSCs. This is in stark contrast to haematopoiesis in healthy individuals who do not develop comparable oligoclonality until the final decades of life^3,5^.

**Fig. 6:**
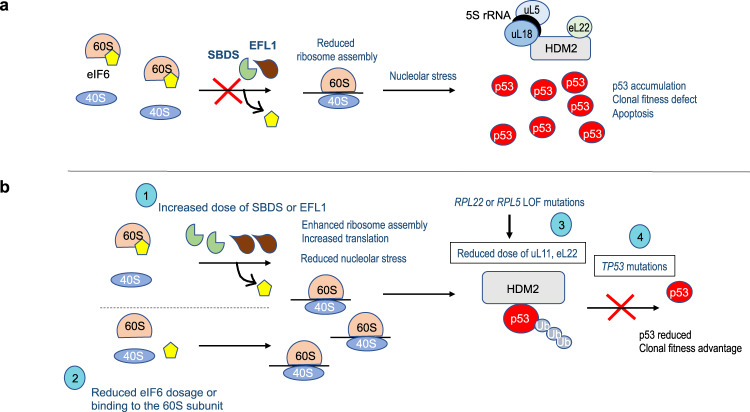
*TP53* and the nucleolar surveillance pathway as targets for convergent somatic mutation. **a** Defective germline ribosome assembly in SDS promotes nucleolar stress through inhibitory binding of the 5S RNP complex (consisting of the 5S rRNA, uL5, encoded by *RPL11* and uL18, encoded by *RPL5*) to the nuclear E3 ligase HDM2 (enhanced by eL22, encoded by *RPL22*), promoting p53 accumulation and apoptosis^22^. **b** Convergent evolution of somatic mutations restores ribosome homoeostasis, favouring HDM2-dependent p53 ubiquitination and degradation, through multiple independent somatic genetic rescue events including: (1) increased dose of SBDS or EFL1 proteins (2) reduced eIF6 dosage or eIF6 binding to the 60S subunit; (3) disrupted inhibitory binding of HDM2 to p53 through mutations in *RPL5* and *RPL22* ^50^; (4) *TP53* mutations. Ub ubiquitin.

We posit that the repertoire of target gene mutations under selective pressure in SDS highlights several routes to improved clonal fitness (Fig. 6a, b). Mutations may be directly compensatory by increasing the gene dosage of either *SBDS* or *EFL1* (Fig. 6b, ‘1’). The increase in *EFL1* gene copy number due to the structural chromosome 15 aberration observed in this study (in the context of germline *SBDS* mutations) is distinct from the somatic *EFL1* copy number changes due to uniparental disomy that have been reported in SDS cases caused by germline *EFL1* mutations^17^. Adaptive somatic mutations that reduce the *EIF6* gene copy number, reduce the level of eIF6 protein or alter its 60S subunit binding activity, may lower the requirement for functional SBDS and EFL1 to release eIF6 from the 60S ribosomal subunit (Fig. 6b, ‘2’). Each of these routes might be expected to help restore ribosome homoeostasis. However, it is important to note that this improved fitness is in the context of the SDS disease state and although somatic *SBDS* gene dosage may be increased, this involves a hypomorphic variant allele that is expected to be only partially restorative. Similarly, alteration of *EIF6* gene copy or alteration of function is restorative, but in context of SBDS deficiency. Thus, neither restoration, nor other noted changes would necessarily rescue all the functional deficiencies of SDS hematopoietic cells.

Defective ribosome assembly activates p53-induced apoptosis via the nucleolar surveillance pathway. Ribosomal proteins uL18 (encoded by *RPL5*) and uL5 (encoded by *RPL11*) bind to the 5S RNA to form the pre-ribosomal 5S ribonucleoprotein (5S-RNP) complex that inhibits the E3 ubiquitin ligase HDM2 to stabilise the p53 protein^22^ (Fig. 6a). eL22 (encoded by *RPL22*) may also inhibit the HDM2-p53 circuit^50^. Thus, somatic mutations in regulators of the nucleolar signalling pathway such as *RPL5* and *RPL22* may disrupt nucleolar stress-induced stabilisation of p53 (Fig. 6c, ‘3’) or mutations in *TP53* itself may allow cells to survive despite ongoing impairment of protein synthesis (Fig. 6b, **‘**4’). The identification of recurrent mutations in *RPL5* and *RPL22* in this study may reflect the use of whole-genome sequencing versus the exome sequencing approach used by Kennedy et al.^18^. Although more speculative, mutations in the evolutionarily conserved splicing factor *PRPF8* may also disrupt the splicing of components of the p53-HDM2 axis including uL18, uL5 or p53 itself^51^. The recurrent *CSNK1A1* mutations identified by Kennedy et al.^18^ (but not in this study) may potentially reflect the role of casein kinase in 40S ribosomal subunit maturation^52^.

Somatic mutation facilitates driver mutation entry, providing a substrate for clonal selection. Estimates of HSC number in healthy humans suggest that 50,000–200,000 uniquely identifiable and actively contributing HSCs are present in adulthood^1,3^. With individual HSCs accumulating ~15 somatic mutations/year^1,3^, one would expect ~1–3 million somatic mutations to enter the HSC pool per year, of which ~10,000–30,000 would be expected to land in coding sequence every year. This number of expected mutations is still greater than the coding footprint of many genes, making it plausible that a nonsynonymous somatic mutation could land in a single gene, such as *TP53* (CDS length 1182 bp), in one HSC within the stem cell pool every year. Thus, opportunities for stochastic somatic mutation of genes in HSCs are very likely to be significantly higher than appreciated, resulting in extensive somatic mosaicism within our HSC pool. This may explain the high prevalence and recurrence of driver mutations in individuals with SDS when there is strong selection facilitating their clonal expansion post acquisition. An interesting hypothesis to test is whether, the numbers of somatic mutations observed in SDS cases at young age might, at least in part, reflect the inability of the immune system in SDS to clear rogue cells with driver mutations.

The stochastic nature of somatic driver mutation acquisition, from early life, may also explain the considerable heterogeneity in clinical phenotype, even amongst siblings with SDS with the same germline genotype^13^. Evidence of strong clonal selection was not captured in all individuals with SDS, as three individuals in the cohort did not harbour detectable clonal expansions or driver mutations (Fig. 2). SDS1 was the youngest individual in our cohort (4.2 years) and thus may have had less time to acquire driver mutations and clonal expansions. Alternatively, clonal expansions may have been missed due to a combination of the small sample size (*n* = 18 colonies) and greater HSC clonal diversity expected in younger individuals^3^. SDS3 was the only individual without the *SBDS* c.183_184TA > CT allele, instead carrying two *SBDS* germline mutations (c.258 + 2T > C, c.258 + 1G > C) that disrupt the intron 2 donor splice site^7^. SDS9 was one of the older individuals in our cohort (26.2 years) and similar individuals lacking driver mutations were also observed by Kennedy et al.^18^. In future, it will be interesting to determine whether individuals who progress to marrow aplasia represent a specific subset of SDS disease evolution where driver mutation mediated clonal expansion has been insufficient to mitigate the bone marrow failure phenotype.

Although our study is limited by the small cohort of individuals included and the lack of longitudinal sampling, technologies such as single molecule sequencing^39,53^ will bypass the requirement for clonal expansions for the detection of driver mutations which may help elucidate the complete spectrum of target genes that provide fitness in SDS. Characterisation of different congenital bone marrow failure disorders may also help us understand the nature of the selective advantage provided by driver mutations associated with clonal haematopoiesis occasionally observed in this and an earlier study^18^.

Clinically, individuals with SDS merit close monitoring of emergent clones via regular extended gene sequencing of blood, given the large number of monoallelic *TP53* clonal expansions that many have, each potentially serving as a substrate for clonal evolution to aggressive disease. Our study and others^18^ suggest that a key mechanism of transformation in SDS is the acquisition of biallelic mutated *TP53*. Given the very rapid clonal outgrowth and genomic evolution observed, consideration for early therapeutic intervention, such as bone marrow transplantation, may be warranted given the poor prognosis associated with *TP53-*mutated myeloid cancers^35,54–58^ and transformed disease in SDS^36^.

## Methods

### SDS samples

Our research complies with all relevant ethical regulations. Individuals with SDS (*n* = 10) were prospectively involved in the study following full Research Ethics Committee approval and consent (NHS Research Ethics Committee approvals 07/MRE05/44 (Cambridge South), 11/LO/0512 (London Riverside), 12/EE/0478 (East of England)). Each individual was sampled at one time point, with the exception of SDS5, who was sampled at two time points. Material included peripheral blood and/or bone marrow and buccal swabs for each individual. Sample collections, initial sample processing and sample banking was performed by the Cambridge Blood and Stem Cell Biobank with appropriate NHS Research Ethics committee approval (18/EE/0199 (East of England). Patients provided written informed consent to use the materials for the research undertaken here and publish the results without compensation.

### HSPC phenotyping

Flow cytometric immunophenotyping of HSPCs was done on stored frozen viable mononuclear cells (MNCs) from PB and/or BM samples obtained from individuals with SDS aged 4–33 years or from healthy/non-SDS donors aged 29–32 years (STEMCELL Technologies). MNCs from healthy (non-SDS) donors were stained with antibodies: CD3-FITC (clone HIT3a, BD #555339; dilution 1:500), CD90-PE (clone 5E10, Biolegend, #328114; 1:33), CD49f-PECy5 (clone GoH3, BD #551129; 1:100), CD38-PECy7 (clone HIT2, Biolegend, #303516; 1:100), CD33-APC (clone WM53, BD #571817; 1:200), CD19-A700 (clone HIB19, Biolegend #302226; 1:300), CD34-APCCy7 (clone 581, Biolegend #343514; 1:100), CD45RA-BV421 (clone HI100, Biolegend #304130; 1:100), and Zombie Aqua (Biolegend #423101; 1:2000). MNCs from individuals with SDS were stained with the following antibodies: CD38-FITC (clone HIT2, BD #555459; 1:12.5), CD34-PE-Cy7 (clone 8G12, BD #348811; 1:33), CD10-BV605 (clone HI10a, Biolegend #312222: 1:33), CD45RA-V450 (clone HI30, BD #560367; 1:100), CD90 APC (Clone 5E10, Biolegend #328110; 1:50), CD3-APC-Cy7 (clone SK7, Biolegend #344818; 1:50), and CD19-APC-Cy7 (clone HIB19, Biolegend #302218, 1:50). After gating for live singlets (7AAD or Zombie negative) and excluding CD3/CD19 positive cells, bulk CD34 positive progenitors were gated (Supplementary Fig. 1).

### In vitro expansion of haematopoietic colonies from mononuclear cells or HSPCs

Previous studies have shown that mutant clonal fractions are equivalent when stem cells or progenitors are sourced from peripheral blood or bone marrow^1,3^. PB or BM samples were collected in Lithium-Heparin (LiHep) tubes, MNCs were isolated by density gradient centrifugation, and RBCs were lysed in NH_4_Cl. Cells from the MNC fraction (or CD34^+^ cells for one individual) were plated into MethoCult H4435 (STEMCELL) for in vitro culture and clonal expansion (colony-forming cell (CFC) assay) using a wide range of cell dilutions in order to ensure appropriate seeding density for picking single-cell-derived colonies. After 2–3 weeks in the CFC assay, individual haematopoietic colonies, were picked into PBS or ProteinaseK buffer (Arcturus Picopure DNA Extraction Kit, Applied Biosystems) and stored at −20 °C for subsequent whole-genome DNA sequencing (WGS) (Fig. 1). In two samples, single-cell liquid cultures were initiated with single lin^−^CD34^+^CD38^+^CD90^−^ cells using the following antibodies: CD38-FITC (Clone HIT2, BD, San Jose, CA, USA; #555459; 1:12.5), CD34 PerCp-Cy5.5 (Clone 581, Biolegend #343522; 1:33, San Diego, USA, CD90-APC (Clone 5E10, Biolegend #328114; 1:33), after pre-enrichment for CD34^+^ cells (EasySep Human CD34 Positive Selection Kit, STEMCELL). Single HSPCs (lin^−^CD34^+^CD38^+^CD90^−^) were flow-sorted using a BD Influx sorter and cultured in StemSpan supplemented with cytokines and recombinant growth factors SCF, FLT3L, IL3 and IL6 (cc100, STEMCELL).

### DNA extractions

DNA from picked CFC colonies were extracted using the Arcturus Picopure DNA extraction kit (Applied Biosystems). DNAeasy kit (Qiagen) was used for extraction from CFC colonies picked into PBS. DNA from buccal swabs was isolated using QIAmp DNA micro kit (Qiagen Cat. 56304).

### Whole genome sequencing of colonies

Individual colonies underwent whole-genome sequencing to identify somatic single nucleotide variants (SNVs), germ line variants, insertions/deletions and structural variants. We generated 150 bp paired-end sequencing reads using Illumina X Ten machines, resulting in a mean coverage of ~20x per sample. The sequences were aligned to the human reference genome GRCh37d5 using the BWA-MEM algorithm^59,60^. Following removal of colonies due to low sequencing depth (less than 6x) and low clonality (median variant allele frequency of less than 0.4), 323 colonies (range of 10–100 per individual, mean of 32 per individual) were taken forward for subsequent analysis.

### Somatic mutation identification and filtering

Single nucleotide variants (SNV) were identified using CaVEMan^61^ for each colony by comparison to an in-silico unmatched sample (PD37Is). CaVEMan was run with the “normal contamination of tumour” parameter set to zero, and the tumour/normal copy numbers set to 5/2. In addition to standard filters, reads supporting an SNV had to have a median BWA-MEM alignment Score ≥ 140 and less than half of the reads clipped. Filtering designed for quality control following processing through the Sanger low-input sequencing pipeline was also applied^62^. The use of the unmatched normal meant that this process called both somatic and germline SNVs. The removal of germline SNVs and artefacts of sequencing required further filtering. As published^4^, we used pooled information across colonies and read counts from a matched germline WGS buccal sample to ensure that genuine somatic variants that may have been present in the germline sample, either as embryonic variants or due to tumour-in-normal contamination were also identified. Short insertions and deletions were called using cgpPindel^63^ with the standard WGS cgpPindel VCF filters applied, except the F018 Pindel filter was disabled as it excludes loci of depth <10. Copy-number aberrations (CNA) were identified using ASCAT^64^ with comparison to a matched normal sample. The union of colony SNVs and insertion–deletions (indels) was then taken and reads counted across all samples belonging to the individual (colonies and buccal samples) using VAFCorrect.

Structural variants (SVs) were called by BRASS^65^. We removed artefacts from the SV calls using AnnotateBRASS (https://github.com/MathijsSanders/AnnotateBRASS^66^) with default settings.

### Creating a genotype matrix

The genotype at each locus within each sample was either 1 (present), 0 (absent) or NA (unknown). We inferred the genotype in a depth sensitive manner. We assumed the observed mutant read count for a colony at a given site was MTR ~ Binomial(*n* = Depth, *p* = expected VAF), if the site was mutant, and MTR ~ Binomial(*n* = depth, *p* = 0.01), if the site was wild-type. The genotype was set to the most likely of the two possible states provided one of the states was at least 20 times more likely than the other. Otherwise the genotype is set to missing (NA). The expected VAF was usually 0.5 for autosomal sites, but for chromosomes X, Y and CNA sites, it was set to 1/ploidy. For loss-of-heterozygosity (LOH) sites, the genotype was overridden and set to missing if it was originally 0.

### Phylogenetic tree topology

We constructed phylogenetic tree topologies using maximum parsimony with MPBoot^67^. The inputs for MPBoot were the binary genotype matrices with missing values per individual. Only SNVs were used to infer the topology, but both SNVs and indels were subsequently assigned to the branches of phylogenetic trees.

### Mutation assignment and branch length adjustment

Mutations were then assigned to the tree in a depth sensitive manner using treemut (https://github.com/nangalialab/treemut^4^) with mutations being hard-assigned to the highest probability branch. Furthermore, branch lengths were adjusted for the branch specific SNV detection sensitivity^4^, where the sensitivity of detection of fully clonal SNV variants was directly estimated from the per colony sensitivity for detecting germline heterozygous SNVs together with a multiplicative correction for the clonality (VAF) of the colonies. In calculating mutation burden and branch lengths copy number regions that are present in any colony in an individual are uniformly masked out in all colonies for that individual and then the overall mutation burden is scaled back up by the reciprocal of 1-expected number of mutations in the masked region.

In addition to SNVs and indels, colonies exhibited a variety of LOH and CNA events. These events were curated as being present or absent in each of the colonies giving an event genotype vector similar to that obtained for SNVs and indels. Once the tree topology was inferred using the SNV genotypes, the branches that exactly matched the event genotype were identified and the event assigned to the corresponding branch.

### Timing branches

Given the linear accumulation of somatic mutations with age, we can infer the time point in life when driver mutations in phylogenetic trees had occurred. Branches at the top of a tree comprise mutations acquired at a young age, with branches lower down representing mutations arising later in life.

We have developed a formal model-based method rtreefit (https://github.com/nangalialab/rtreefit) for converting trees where branch lengths are expressed in molecular time (i.e. number of mutations) into trees where the branch lengths are expressed in units of time (years)^4^. In brief, the method jointly fits a single constant mutation rate (i.e. number of SNVs accumulated per year) and absolute time branch lengths using a Bayesian per individual tree-based model under the assumption that the number of observed mutations assigned to a branch is Poisson distributed with Mean = Branch Duration × Sensitivity × Mutation Rate, and subject to the constraint that the root to tip duration is equal to the age at sampling. Additionally, the method accounts for an elevated mutation rate during embryogenesis by assuming an excess mutation rate through development.

The rtreefit algorithm was run with 4 chains and 20,000 iterations per chain.

### Detection of driver mutations in WGS data

We searched specifically for hotspot driver mutations, copy number changes and rearrangements in 35 genes known to be associated with haematological malignancy^35^ and clonal haematopoiesis^33,34^ (*ASXL1, BCOR, CALR, CBL, CSF3R, CUX1, DNMT3A, EZH2, GATA2, GNAS, GNB1, IDH1, IDH2, JAK2, KIT, KRAS, MLL3, MPL, NF1, NFE2, NRAS, PHF6, PPM1D, PTPN11, RB1, RUNX1, SETBP1, SF3B1, SRSF2, SH2B3, STAG2, TET2, TP53, U2AF1, ZRSR2*) as well as in the recurrently mutated genes identified in SDS. We identified somatic mutations under positive and negative selection using dNdScv^68^.

### Mutational signature analysis

We characterised mutational profiles present in our dataset by performing signature extraction with *hdp* (https://github.com/nicolaroberts/hdp) without any signatures as prior and with no specified grouping of the data. In order to avoid double counting, mutations shared among colonies were randomly assigned to one colony. *hdp* identified the presence of 2 mutational signatures, one with strong similarity to Cosmic signatures SBS1^42^ (cosine similarity ≥0.95) and one with strong similarity to SBSblood^1,2,41^ (cosine similarity ≥0.91). We then estimated the proportion of SBS1 and SBSblood mutational signatures present in each colony using the programme *sigfit* ^69^.

### SDS8 mutation burden and comparison to other individuals

We define the overdispersion in mutation burden as the ratio of the expected burden variance to Poisson variance. We estimate the overdispersion as a function of age using data from healthy/non-SDS blood single-cell-derived colonies reported in Mitchell et al.^3^. The within-individual overdispersion at each time point is estimated as the sample mutation burden variance divided by the sample mean mutation burden. The log overdispersion was then modelled using a linear model with age as the explanatory variable. The estimated overdispersion at age 25 is 2.24 (1.85–2.73). For the purposes of assessing the statistical significance of the apparently high SDS8 mutation burden we conservatively account for the very high degree of shared history of the SDS8 colonies by regarding the clade as a single-cell with burden given by the mean burden, and then assess the probability of observing such an extreme mutation burden (*n* = 905) under the null hypothesis that mutations were accrued according to a negative binomial distribution with a mean equal to the expected number of mutations (*n* = 496) and a variance that is 2.24 times the mean.

### SDS8 timing of rapid growth and selection

We assume that there is a single transformation event that switches on a mutational process that is responsible for the distinct signature profile (transformation signature) observed in the expanded clade (Supplementary Fig. 4). We then estimate the timing of this event as the age that corresponds to the number of trunk mutations that can be attributed to the composite signature profile of normal SDS haematopoiesis (SDS6, SDS7 and SDS9) (composite age-matched SDS signature). The number of composite age-matched SDS signature trunk mutations accrued is estimated by using the R package sigfit^69^ to decompose the trunk SNVs into contributions from the composite age-matched SDS signature and the transformation signature. We then estimate the corresponding age using Approximate Bayesian Computation with the rejection method, requiring that the number of substitutions acquired since birth follows a negative binomial distribution with a mean = age at transformation × mutation rate, and variance set to 2.24 times the mean (see section above). The mutation rate itself is drawn from a normal distribution with mean 15.1 and variance of 1. The unconditional age estimate uses a uniform prior age range of 0–100 years, whereas the conditional age estimate uses a uniform prior range of 0–25 years. We estimate an ultrametric tree using rtreefit^4^ (with age of transformation constrained to the lower bound of the 95% CI for the conditional age estimate (19.3 years). The phylofit method^3^ was then used to estimate the rate of clone growth using the timing and pattern of coalescences. Full details can be found in https://github.com/nangalialab/ShwachmanDiamond.

### Plasmid generation

cDNA for human eIF6 WT carrying a C-terminal FLAG-tag was generated by PCR using the Phusion High-Fidelity PCR kit (NEB). The PCR product was then inserted into pcDNA3.1 (Thermo Fisher Scientific) using the BamHI/XhoI sites, generating plasmid pEIF6-WT-FLAG). Site-directed mutagenesis was performed to generate the eIF6 M1T mutant (plasmid pEIF6-M1T-FLAG) using the Phusion High-Fidelity PCR kit (Thermo Fisher Scientific). Primers were as follows (5’ to 3’):

eIF6-WT-Fw,TACTGGATCCATGGCGGTCCGAGCTTCGTTC

eIF6-WT-FLAG-Rev,AGTACTCGAGTCACTTGTCGTCATCGTCTTTGTAGTCGGTGAGGCTGTCAATGAGGGAATC) eIF6-M1T-Fw,CGGATCCACGGCGGTCCGAGCTTCGTTCGAGAACAA

eIF6-M1T-Rev, GGACCGCCGTGGATCCGAGCTCGGTACCAAGCTTAA

### Immunoblotting of human and *Drosophila* cell extracts

HEK293T cells (Sigma, 12022001) were grown in a 12-well dish to ~80% confluence followed by plasmid transfection using lipofectamine 2000 (Thermo Fisher Scientific) for 24 h. The cells were washed in 1x PBS and lysed in 0.5% NP-40 for 30 min on ice. The lysates were centrifuged at 21,130 × *g* for 10 min and the supernatant mixed with 50 mM DTT and 4x NuPAGE LDS sample buffer (Thermo Fisher Scientific) to 1x. Samples were incubated at 70 °C for 10 min. The proteins were separated in a NuPAGE 4–12% Bis-Tris gel (Thermo Fisher Scientific) in 1x MES running buffer (Formedium) prior to transfer to nitrocellulose membrane using the iBlot 2 (Thermo Fisher Scientific) system. The membrane was blocked with 5% (w/v) milk dissolved in PBST buffer (1x PBS with 0.1% [v/v] Tween 20) for 1 h. Human proteins were visualised using anti-FLAG (Sigma, #F7425, 1:5000 dilution), anti-eIF6 (GenTex, #GTX117971) and anti-actin antibodies (Sigma, #A2066), both at 1:1000 dilution. Anti-rabbit IgG HRP-linked antibody (Cell Signalling, #7074; 1:5000) was used as the secondary antibody. Blots were developed with the Western Chemiluminescent HRP substrate (Immobilon) and visualised using the Chemidoc™ MP (Bio-Rad) imaging system. Analysis was performed using Image Lab software v6.0.1 (Bio-Rad).

*Drosophila* third instar larvae (typically 15 larvae) were collected, washed with PBS, homogenised in lysis buffer (20 mM HEPES pH 7.4, 50 mM KCl, 2.5 mM MgCl_2_, 0.5% (v/v) IGEPAL® CA-630 (Sigma, #I8896), 0.5% (w/v) Sodium deoxycholate (Sigma, #30970) with complete EDTA-free protease inhibitors (Roche) and incubated for 15 min on ice. Lysates were cleared in a microcentrifuge at 20,000 × *g* for 10 min at 4 °C. Equal amounts (typically 10 µg) of total protein were loaded and separated using SDS-PAGE for immunoblotting. *Drosophila* proteins were visualised using anti-Gapdh (Merck #G9545, 1:20,000 dilution), anti-eIF6 (GeneTex, #GTX117971, 1:1000 dilution) and anti-FLAG antibodies (Abcam, 1:20,000 dilution). Secondary antibodies were all used at 1:10,000 dilution: anti-mouse IgG, HRP-conjugated (Sigma-A5287), anti-rabbit IgG, HRP-conjugated (Cell Signalling 7074), anti-goat IgG, HRP-conjugated (Santa Cruz, sc-2020) antibody. Blots were developed with the SuperSignal™ West Pico PLUS Chemiluminescent Substrate (Thermo Fisher, #34580), and visualised using a Chemidoc™ MP (Bio-Rad) imaging system. Analysis was performed using Image Lab software v6.0.1 (Bio-Rad).

### *Drosophila melanogaster* strains and genetics

Flies were maintained using standard culture techniques. All crosses were performed at 25 °C. Fly strains and genotypes are described in Supplementary Tables 2 and 3. The *Drosophila* lines *Sbds*^*P*^, *UAS-EIF6-FLAG*, *UAS-EIF6-R96W-FLAG*, *UAS-EIF6-N106S-FLAG* are described in Supplementary Table 3. To generate the *UAS-EIF6-I58T-FLAG* transgenic line, the coding sequence for *Drosophila EIF6* (NM_145105) was amplified by PCR from *Drosophila* larval cDNA^18^. The variant *EIF6*^*I58T*^ was generated by PCR site-directed mutagenesis and sub-cloned into pTWF (The *Drosophila* Gateway vector collection) to generate plasmid pUAS-EIF6-I58T-FLAG for microinjection. The transgenic *pUAS-EIF6-I58T-FLAG* line was generated by P element–mediated germline transformation into a *w* ^*1118*^ strain by BestGene Inc. Oligonucleotide primers used to generate the *Drosophila* strains were as follows (5’ to 3’): EIF6-F: CACCATGGCTCTACGCGTCC; EIF6-R: GGACATGTCCTCGATGAGGGC; EIF6-I58T-F: CTGCCGGACAATCGGCCGCC; EIF6-I58T-R: GCCGATTGTCCGGCAGCCG. The da-GAL4 line was used to induce ubiquitous expression of FLAG-tagged eIF6 variants under the UASt promoter.

### Reporting summary

Further information on research design is available in the Nature Portfolio Reporting Summary linked to this article.

### Supplementary information

Supplementary Information

Description of Additional Supplementary Files

Supplementary Datasets 1 and 2

Reporting Summary

### Source data

Source Data

## Data Availability

Raw sequencing data for all whole genomes are available at the European Genome-Phenome Archive (accession number EGAD00001009061. Access to this human data hosted in the EGA is restricted due to sensitivity, and therefore it is managed in line with the Wellcome Sanger Data Sharing Policy. Researchers interested in accessing the data should submit a data access application to Sanger via eDAM2 (https://edam.sanger.ac.uk/). Further details can be found at https://www.sanger.ac.uk/about/edam2-guide/#02-04. When an application is received, a variety of checks are conducted by the data access team, e.g. the applicant’s identity as a bona-fide researcher, their affiliation and the project they describe in their application is in line with any usage restrictions associated with the dataset(s) they have requested. There is no time limit on data access; the data access agreements are perpetual and run until terminated. However, the data access would be associated with (1) a specific project, so can only be used for that project for as long as it runs and (2) the researcher’s institutional email address, so if they change affiliation, they would lose access to the data and would need to re-apply for data access under their new affiliation. Source data are provided with this paper.

## Code availability

A repository of the code for conducting all analyses can be found at https://github.com/machadoheather/somatic_evolution_SDS (10.5281/zenodo.8172028)^70^ and https://github.com/nangalialab/ShwachmanDiamond (10.5281/zenodo.8172581)^71^.
