# Supplementary material for: Convergent somatic evolution commences in utero in a germline ribosomopathy
Source: Nat Commun. 2023 Aug 22;14:5092. doi: 10.1038/s41467-023-40896-5 (PMC10444798; doi:10.1038/s41467-023-40896-5)
Supplement: Supplementary file 1 — Supplementary Information [file 41467_2023_40896_MOESM1_ESM.pdf]

Supplementary Information

Supplementary Figure 1

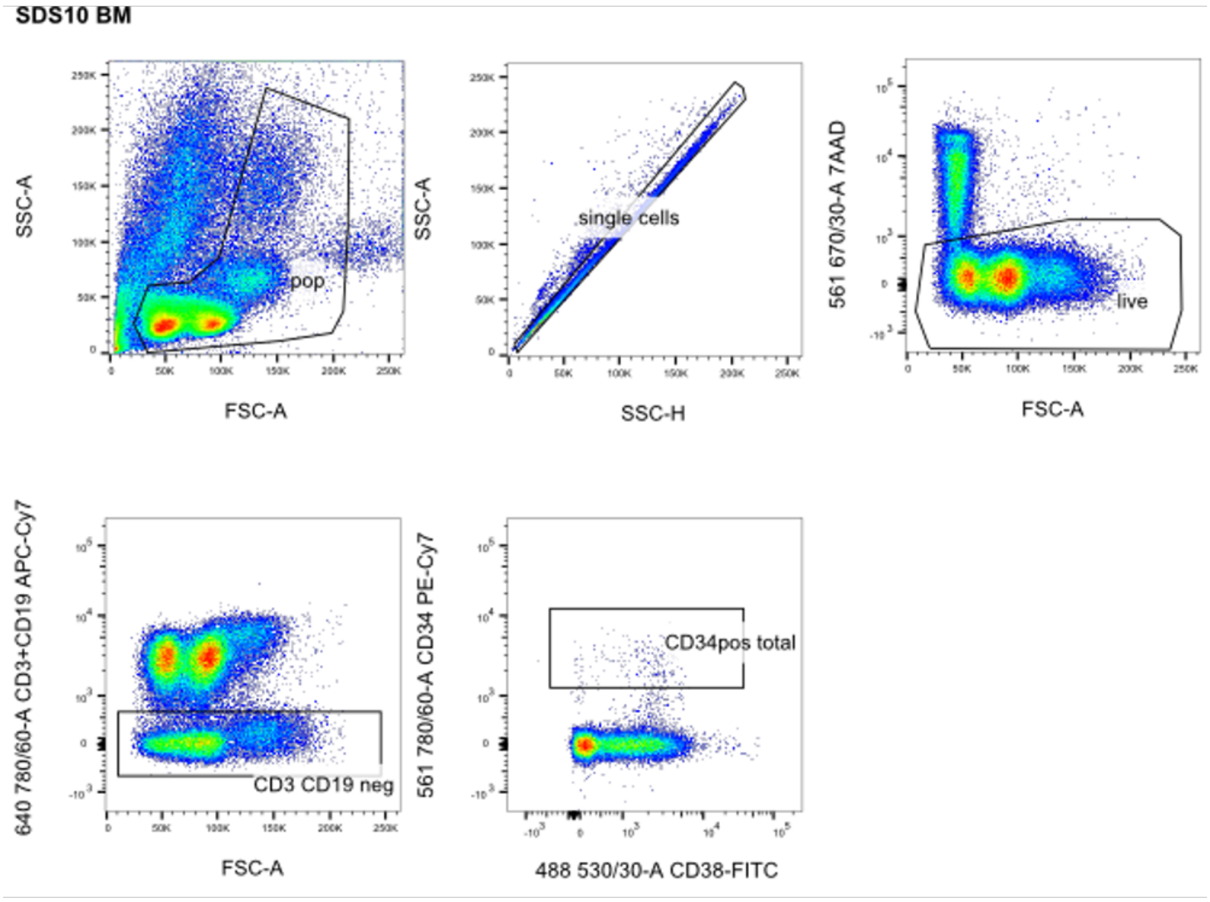

Supplementary Figure 1. Gating Strategy. Flow cytometric evaluation of the frequency of CD34$^+$ blood cell progenitors in frozen viable samples (peripheral blood and/or bone marrow) from individuals with SDS and healthy/non-SDS healthy donors. The gating strategy is shown above which starts with an exclusion of cell debris, gating for single cells followed by live cells (7AAD-negative fraction), with subsequent exclusion of CD3/CD19 positive cells to exclude lymphocytes, followed by gating for CD34-positive progenitors. The plots show an example from one individual (SDS10) bone marrow.

Supplementary Figure 2

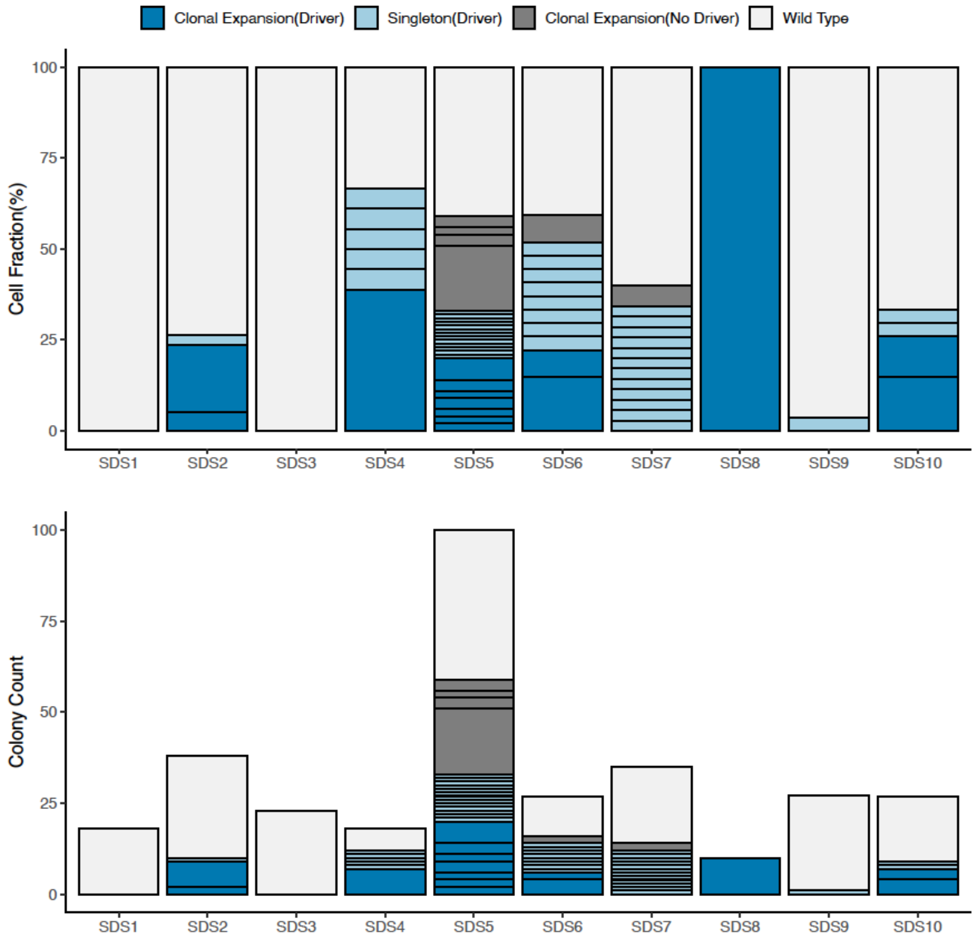

Supplementary Figure 2. Classification of colonies by fraction of colonies (top panel) and colony number (lower panel) according to their status with respect to harbouring a driver mutation or being part of a clonal expansion. Colonies were classed as belonging to clonal expansions with (dark blue) or without (grey) identified driver mutations, single colonies (singletons) harbouring known driver mutations (light blue), and wild type colonies (white) (i.e. those colonies that were not part of clonal expansions and did not harbour a driver mutation). A clonal expansion was defined as any clade with 2 or more members where the most recent common ancestor of the clade occurred after 75 mutations from the top of the phylogenetic trees. Individual expansions and singletons with driver mutations are demarcated by horizontal black lines. Source data are provided as a Source Data file.

Supplementary Figure 3

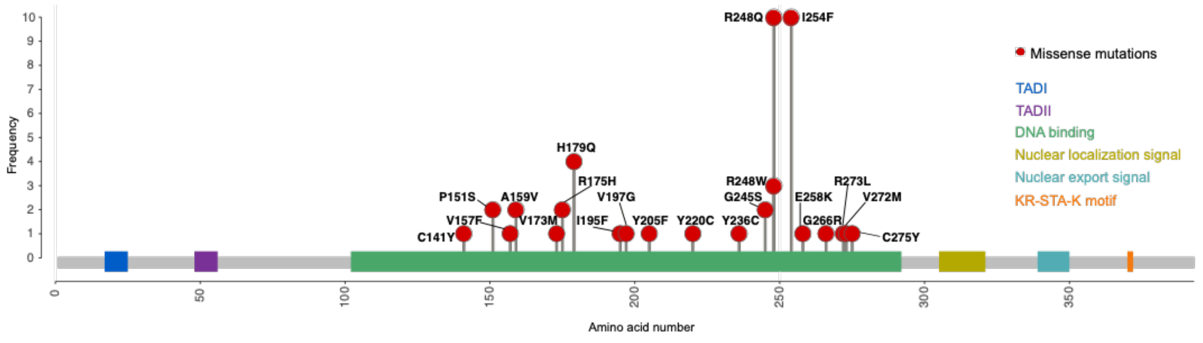

Supplementary Figure 3. Location of somatic mutations affecting *TP53* in individuals with SDS in the study. All mutations (red dots) were missense single nucleotide variants affecting the DNA binding domain (green). Protein domains are coloured by amino acid (x axis) location. Number of mutations observed are shown on the y axis. TAD, transactivation domain.

Supplementary Figure 4

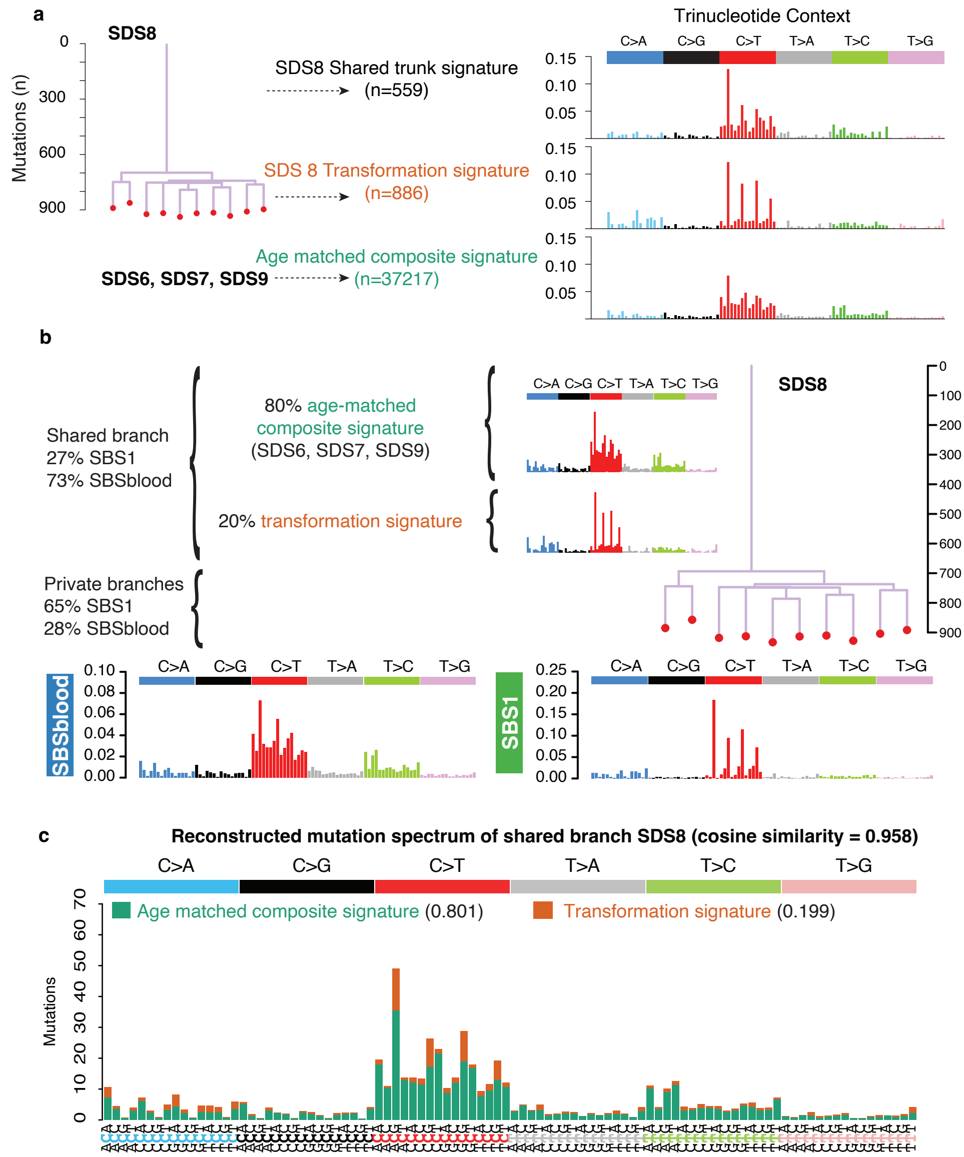

Supplementary Figure 4. Shared and derived mutational profiles in SDS8 genomes. **a,** Mutation spectrum of the shared branch of SDS8 (top panel), private branches of the clonal expansion ("transformation signature", middle panel), and from individuals of a similar age ("age matched composite signature", SDS6, 7, 9) from the study (bottom panel). Number of mutations are shown in brackets. **b,** The trunk of the phylogenetic tree, representing shared mutations, has a lower (27%) SBS1 attribution than private branches (65%), indicating an increased rate of SBS1 mutagenesis during the clonal expansion event. The estimated proportion of the "transformation signature", and the "age matched composite signature" are shown. **c,** Reconstruction of the shared branch of SDS8 using the "age matched composite" and "transformation" mutational signatures using *sigfit*[70]. X axis shows the trinucleotide context of the different mutations.

Supplementary Tables

Supplementary Table 1. Peripheral blood counts at time of initial sampling.

ID	Hb (g/L)	Wbc ($x10^9/L$)	Neuts ($x10^9/L$)	Plts ($x10^9/L$)	MCV (fL)	Retics ($x10^9/L$)
SDS1	110	3.65	0.95	293	78.8	N/A
SDS2	126	4.6	0.67	233	78	N/A
SDS3	122	3.71	1.07	156	79.2	N/A
SDS4	116	5.72	2.63	109	92.3	N/A
SDS5	148	3.1	1.03	121	101.5	49
SDS6	152	5.7	3	190	91.7	58
SDS7	144	3.6	1.29	115	92.5	97.1
SDS8	79	1.2	0.14	21	96.7	45.7
SDS9	160	2.8	0.82	181	94.8	90.3
SDS10	130	2.4	1.03	123	102.2	47.8

N/A, not available

Supplementary Table 2. ***Drosophila*** **genotypes**

Figure	Name	Genotype
**5f, 5g**	Control	*w^{1118};; da-GAL4*
**5f, 5g**	*EIF6/+*	*w^{1118}; UAS-EIF6-FLAG/+; da-GAL4/+*
**5f, 5g**	*EIF6-I58T/+*	*w^{1118}; UAS-EIF6- I58T-FLAG/+; da-GAL4/+*
**5f, 5g**	*EIF6-R96W/+*	*w^{1118}; UAS-EIF6-R96W-FLAG/+; da-GAL4/+*
**5f, 5g**	*EIF6-N106S/+*	*w^{1118}; UAS-EIF6-N106S-FLAG /+; da-GAL4/+*
**5h**	WT	*w^{1118}*
**5h**	*$Sbds^{P/P}$*	*w^{1118}; $Sbds^{P/P}$*
**5h**	*$Sbds^{P/P}$, EIF6/+*	*w^{1118}; UAS-EIF6-FLAG/+; da-GAL4, $Sbds^{P}$/$Sbds^{P}$*
**5h**	*$Sbds^{P/P}$, EIF6-I58T/+*	*w^{1118}; UAS-EIF6-I58T-FLAG/+; da-GAL4, $Sbds^{P}$/$Sbds^{P}$*
**5h**	*$Sbds^{P/P}$, EIF6-R96W/+*	*w^{1118}; UAS-EIF6-R96W-FLAG/+; da-GAL4, $Sbds^{P}$/$Sbds^{P}$*
**5h**	*$Sbds^{P/P}$, EIF6-N106S/+*	*w^{1118}; UAS-EIF6-N106S-FLAG/+; da-GAL4, $Sbds^{P}$/$Sbds^{P}$*

Supplementary Table 3. ***Drosophila*** **strains**

**Name**	**Genotype**	**Source**
Wild type (WT)	w^{1118}	J. Root (University of Cambridge UK)
$Sbds^{P}$	w^{1118}; *PBac{WH}CG8549*^{f01686}*/TM6B, Tb*^{1}	Exelixis (Harvard)
da-GAL4	w^{*}; *P{GAL4-da.G32}UH1*	Bloomington *Drosophila* Stock Centre
UAS-EIF6-FLAG	w^{1118}; *pUAS-EIF6-FLAG*	Alan J Warren
UAS-EIF6-I58T-FLAG	w^{1118}; *pUAS-EIF6-I58T-FLAG*	Alan J Warren
UAS-EIF6-R96W-FLAG	w^{1118}; *pUAS-EIF6-R96W-FLAG*	Alan J Warren
UAS-EIF6-N106S-FLAG	w^{1118}; *pUAS-EIF6-N106S-FLAG*	Alan J Warren
