# Supplementary material for: Convergent somatic evolution commences in utero in a germline ribosomopathy
Source: Nat Commun. 2023 Aug 22;14:5092. doi: 10.1038/s41467-023-40896-5 (PMC10444798; doi:10.1038/s41467-023-40896-5)
Supplement: Supplementary file 2 — Description of Additional Supplementary Files [file 41467_2023_40896_MOESM2_ESM.pdf]

Description of Additional Supplementary files

Supplementary dataset 1: SNV/indels per colony genome with gene annotation

Supplementary dataset 2: Somatic variants in expanded lineages (with and without drivers)
