# Supplementary material for: Convergent somatic evolution commences in utero in a germline ribosomopathy
Source: Nat Commun. 2023 Aug 22;14:5092. doi: 10.1038/s41467-023-40896-5 (PMC10444798; doi:10.1038/s41467-023-40896-5)
Supplement: Supplementary file 4 — Reporting Summary [file 41467_2023_40896_MOESM4_ESM.pdf]

nature portfolio

Corresponding author(s): Alan J Warren
David Kent
Jyoti Nangalia

Last updated by author(s): 10.8.23.

Reporting Summary

Nature Portfolio wishes to improve the reproducibility of the work that we publish. This form provides structure for consistency and transparency in reporting. For further information on Nature Portfolio policies, see our Editorial Policies and the Editorial Policy Checklist.

Statistics

For all statistical analyses, confirm that the following items are present in the figure legend, table legend, main text, or Methods section.

n/a	Confirmed	
☐	☒	The exact sample size (*n*) for each experimental group/condition, given as a discrete number and unit of measurement
☐	☒	A statement on whether measurements were taken from distinct samples or whether the same sample was measured repeatedly
☐	☒	The statistical test(s) used AND whether they are one- or two-sided<br>*Only common tests should be described solely by name; describe more complex techniques in the Methods section.*
☐	☒	A description of all covariates tested
☐	☒	A description of any assumptions or corrections, such as tests of normality and adjustment for multiple comparisons
☐	☒	A full description of the statistical parameters including central tendency (e.g. means) or other basic estimates (e.g. regression coefficient) AND variation (e.g. standard deviation) or associated estimates of uncertainty (e.g. confidence intervals)
☐	☒	For null hypothesis testing, the test statistic (e.g. *F*, *t*, *r*) with confidence intervals, effect sizes, degrees of freedom and *P* value noted<br>*Give P values as exact values whenever suitable.*
☐	☒	For Bayesian analysis, information on the choice of priors and Markov chain Monte Carlo settings
☒	☐	For hierarchical and complex designs, identification of the appropriate level for tests and full reporting of outcomes
☒	☐	Estimates of effect sizes (e.g. Cohen's *d*, Pearson's *r*), indicating how they were calculated

Our web collection on statistics for biologists contains articles on many of the points above.

Software and code

Policy information about availability of computer code

Data collection

No software was used for data collection.

Data analysis

FACS analyis: Data acquisition was performed using FACSDiva software (BD Biosciences) v8.

Open source programs used (also stated in manuscript): BWA v0.7.17, CaVEMan v1.11.2, Pindel v3.1.2, BRASS v6.1.2, ASCAT 4.0.1, R v4.0.2, AnnotateBRASS v3, SigProfiler 1.0.0, Telomerecat v4.0.2, FlowJo v10.7.1, FCS Express v7.10.0007, Rstudio v1.4.1073.

R packages used: hdp v0.1.5, sigfit v2.2., Rsamtools v2.2.3, MASS v7.3.57, GenomicRanges v1.38.0, plyr v1.8.5, ggplot2 v3.2.1, foreach v1.4.8, doParallel v1.0.16, reshape2 v1.4.3, sigfit v2.2, stringr v1.4.0, dplyr v0.8.4, RColorBrewer v1.1.2, BSgenome.Hsapiens.UCSC.hg19 v1.4.3, grid v3.5.1, gridExtra v2.3, ggpubr v0.2.4, tidymv v3.2.0, GenomicFeatures v1.42.1, nrmisc v, tidyverse v1.3.0, magrittr v1.5, rtracklayer v1.50.0, BSgenome.Hsapiens.1000genomes.hs37d5 v0.99.1, cowplot v1.0.0

Custom code made available (also stated in manuscript): https://github.com/machadoheather/somatic_evolution_SDS and https://github.com/nangalialab/ShwachmanDiamond

No commercial software used.

Image Lab software v6.0.1 (Bio-Rad), Adobe Photoshop 2022, PRISM 8 (Graph Pad). Single-nucleotide substitutions (SNV) were called using the CaVEMan (Cancer Variants through Expectation Maximization) algorithm, version 1.15.1 (https://github.com/cancerit/CaVEMan). Small insertions and deletions were called using the Pindel algorithm as implemented in the cgpPindel workflow, version 3.5.0 (https://github.com/cancerit/cgpPindel). Copy number variants were called using the ASCAT algorithm as

implemented in the ascatNgs workflow, version 4.5.0 (https://github.com/cancerit/ascatNgs). Mutational signatures analysis was performed using sigFit 2.1 (https://github.com/kgori/sigfit).
Allele counts at SNV and Indel sites were carried out using vafCorrect (https://github.com/cancerit/vafCorrect). Telomere lengths were estimated using telomerecat, version 3.2 (https://github.com/cancerit/telomerecat). Mutations were mapped to phylogenetic branches using treemut (https://github.com/nangalialab/treemut). Temporal branch lengths and were inferred using rtreefit (https://github.com/nangalialab/rtreefit); Other analyses were carried out using custom R scripts, these are in a private Github repository (https://github.com/nangalialab/ShwachmanDiamond) which will be made publicly accessible prior to publication and in the meantime the contents of the repository are made available as a zip file.

Work flow figure (Figure 1.a) was designed by the authors in house using software programmes Inkscape (version 1.1) and Microsoft Powerpoint v16.66.1.

For manuscripts utilizing custom algorithms or software that are central to the research but not yet described in published literature, software must be made available to editors and reviewers. We strongly encourage code deposition in a community repository (e.g. GitHub). See the Nature Portfolio guidelines for submitting code & software for further information.

Data

Policy information about availability of data

All manuscripts must include a data availability statement. This statement should provide the following information, where applicable:

- Accession codes, unique identifiers, or web links for publicly available datasets
- A description of any restrictions on data availability
- For clinical datasets or third party data, please ensure that the statement adheres to our policy

Sequence data that support the findings of this study have been deposited in the European Genome-Phenome Archive (https://www.ebi.ac.uk/ega/home), accession EGAD00001009061.
Sequences were aligned to the human reference genome GRCh37d5 using the BWA-MEM algorithm (Li, H. Aligning sequence reads, clone sequences and assembly contigs with BWA-MEM. arXiv:1303.3997 [q-bio] (2013); Li, H. & Durbin, R. Fast and accurate short read alignment with Burrows-Wheeler transform. Bioinformatics 25, 1754–1760 (2009)).

Raw sequencing data for all whole genomes are available at the European Genome-Phenome Archive (accession number EGAD00001009061, [https://ega-archive.org/datasets/EGAD00001009061]. Access to this human data hosted in the EGA is managed in line with the Wellcome Sanger Data Sharing Policy. Researchers interested in accessing the data should submit a data access application to Sanger via eDAM2 (https://edam.sanger.ac.uk/). Further details can be found at https://www.sanger.ac.uk/about/edam2-guide/#02-04. When an application is received, a variety of checks are conducted by the data access team, eg. the applicant's identity as a bona-fide researcher, their affiliation and the project they describe in their application is in line with any usage restrictions associated with the dataset(s) they have requested. There is no time limit on data access; the data access agreements are perpetual and run until terminated. However, the data access would be associated with 1) a specific project, so can only be used for that project for as long as it runs and 2) the researcher's institutional email address, so if they change affiliation, they would lose access to the data and would need to re-apply for data access under their new affiliation.
Source data are provided with this paper.

Human research participants

Policy information about studies involving human research participants and Sex and Gender in Research.

Reporting on sex and gender	Buccal swabs,blood and/or bone marrow samples were obtained form 10 individuals diagnosed with Shwachman-Diamond syndrome. Ages are shown in Figure 2. Gender was anonymised.
Population characteristics	Samples were obtained from patients with Shwachman-Diamond syndrome attending hospital out-patient clinics. with informed consent to use the materials for the research undertaken here and publish the results without compensation. Ages are shown in Figure 2. SDS donor age range was 4-33, age of healthy bone marrow donors was 29-32 years.
Recruitment	Individuals with SDS (n=10) were prospectively included from UK hospitals following full Research Ethics Committee approval and consent.<br>We studied 10 individuals with SDS aged 4-33 years, who harboured biallelic germline loss-of-function mutations in the SBDS gene. We undertook whole genome sequencing of 323 individual single cell-derived colonies seeded from haematopoietic stem and progenitor cells (HSPC), to a mean depth of 20x reads, together with matched buccal swab DNA as a germline reference, in all individuals. In total, we identified 118,564 single nucleotide variants, 6287 small insertions and deletions, 74 structural variants and 5 chromosomal copy number aberrations across the cohort. This sample size is sufficient to justify all the conclusions that we wish to draw.
Ethics oversight	Our research complies with all relevant ethical regulations. Individuals with SDS (n=10) were prospectively involved in the study following full Research Ethics Committee approval and consent (NHS Research Ethics Committee approvals 07/MRE05/44 (Cambridge South), 11/LO/0512 (London Riverside), 12/EE/0478 (East of England)). Each individual was sampled at one time point, with the exception of SDS5, who was sampled at two time points. Material included peripheral blood and/or bone marrow and buccal swabs for each individual. Sample collections, initial sample processing and sample banking was performed by the Cambridge Blood and Stem Cell Biobank with appropriate NHS Research Ethics committee approval (18/EE/0199 (East of England). Patients provided informed consent to use the materials for the research undertaken here and publish the results without compensation.

Note that full information on the approval of the study protocol must also be provided in the manuscript.

Field-specific reporting

Please select the one below that is the best fit for your research. If you are not sure, read the appropriate sections before making your selection.

☒ Life sciences ☐ Behavioural & social sciences ☐ Ecological, evolutionary & environmental sciences

For a reference copy of the document with all sections, see nature.com/documents/nr-reporting-summary-flat.pdf

Life sciences study design

All studies must disclose on these points even when the disclosure is negative.

Sample size	For each Drosophila experiment, at least 3 replicates were performed with a minimum sample size of 156 flies. These numbers were chosen to provide adequate statistical power.
Data exclusions	No data were excluded from the analysis
Replication	The number of replicates is stated in the figure legends for each experiment, but minimum of 3 and maximum of 5 replicates were performed.
Randomization	Flies of the same genotype were randomly allocated to experimental groups
Blinding	Experimenters were not blinded as fly genotypes would be clear to any observer.

Reporting for specific materials, systems and methods

We require information from authors about some types of materials, experimental systems and methods used in many studies. Here, indicate whether each material, system or method listed is relevant to your study. If you are not sure if a list item applies to your research, read the appropriate section before selecting a response.

Materials & experimental systems

n/a	Involved in the study	
☐	☒	Antibodies
☐	☒	Eukaryotic cell lines
☒	☐	Palaeontology and archaeology
☐	☒	Animals and other organisms
☒	☐	Clinical data
☒	☐	Dual use research of concern

Methods

n/a	Involved in the study	
☒	☐	ChIP-seq
☐	☒	Flow cytometry
☒	☐	MRI-based neuroimaging

Antibodies

Antibodies used

Antibodies used for flow cytometry (Antibody-Flurophore; Clone; Company; Catalogue number; Dilution; Website link):

CD38-FITC; HIT2; BD; #555459; 1:12.5
https://www.bdbiosciences.com/en-dk/products/reagents/flow-cytometry-reagents/research-reagents/single-color-antibodies-ruo/fitc-mouse-anti-human-cd38.560982

CD34-PE-Cy7; 8G12; BD; #348811; 1: 33
https://www.bdbiosciences.com/en-dk/products/reagents/flow-cytometry-reagents/clinical-diagnostics/single-color-antibodies-asr-ivd-ce-ivd/cd34-pe-cy-7.348811

CD10-BV605; HI10a; Biolegend; #312222: 1:33
https://www.biolegend.com/en-gb/products/brilliant-violet-605-anti-human-cd10-antibody-8579?GroupID=BLG5905

CD45RA-V450; HI30; BD; #560367; 1:100
https://www.bdbiosciences.com/en-nz/products/reagents/flow-cytometry-reagents/research-reagents/single-color-antibodies-ruo/v450-mouse-anti-human-cd45.560367

CD90-APC; 5E10; Biolegend; #328114; 1:33
https://www.biolegend.com/nl-be/products/apc-anti-human-cd90-thy1-antibody-4116,
or BD, 559869, 1:33 https://www.bdbiosciences.com/en-br/products/reagents/flow-cytometry-reagents/research-reagents/single-color-antibodies-ruo/apc-mouse-anti-human-cd90.561971

CD3-APC-Cy7; SK7; Biolegend; #344818; 1:50
https://www.biolegend.com/en-us/search-results/apc-cyanine7-anti-human-cd3-antibody-6940

CD19-APC-Cy7; HIB19; Biolegend; #302218, 1:50
https://www.biolegend.com/nl-nl/products/apc-cyanine7-anti-human-cd19-antibody-1910

CD34-PerCp-Cy5.5; 581; Biolegend; #343522; 1:33
https://www.biolegend.com/en-us/antibodies-and-more/percp-cyanine5-5-anti-human-cd34-antibody-6203

CCD3-FITC; clone HIT3a; #555339; BD; dilution 1:500; https://www.bdbiosciences.com/en-us/products/reagents/flow-cytometry-reagents/research-reagents/single-color-antibodies-ruo/fitc-mouse-anti-human-cd3.561802

CD90-PE; 5E10; #328110; Biolegend; 1:50;
https://www.biolegend.com/en-us/productstab/pe-anti-human-cd90-thy1-antibody-4114?GroupID=BLG5826

CD49f-PE-Cy5; GoH3; #551129; BD; 1:100;
https://www.bdbiosciences.com/en-dk/products/reagents/flow-cytometry-reagents/research-reagents/single-color-antibodies-ruo/pe-cy-5-rat-anti-human-cd49f.551129

CD19-A700; HIB19; #302226; Biolegend; 1:300; https://www.biolegend.com/en-gb/search-results/alexa-fluor-700-anti-human-cd19-antibody-3399?GroupID=BLG10095

CD34-APC-Cy7; 581; #343514; Biolegend; 1:100; https://www.biolegend.com/de-de/products/apc-cyanine7-anti-human-cd34-antibody-6159

Zombie Aqua; NA; #423101; Biolegend; 1:2000; https://www.biolegend.com/en-ie/products/zombie-aqua-fixable-viability-kit-8444?GroupID=BLG2181

CD38-PE-Cy7; HIT2; #303516; Biolegend; 1:100; https://www.biolegend.com/en-us/search-results/pe-cyanine7-anti-human-cd38-antibody-5418?GroupID=BLG10099

CD45RA-BV421; HI100; #304130; Biolegend; 1:100; https://www.biolegend.com/fr-fr/products/brilliant-violet-421-anti-human-cd45ra-antibody-7200

CD33-APC; WM53; #571817; BD; 1:200; https://www.bdbiosciences.com/en-eu/products/reagents/flow-cytometry-reagents/research-reagents/single-color-antibodies-ruo/apc-mouse-anti-human-cd33.561817

Human proteins were visualised using:

anti-FLAG (Sigma, #F7425, 1:5000 dilution)
https://www.sigmaaldrich.com/GB/en/product/sigma/f7425

anti-eIF6 (GenTex, #GTX117971, 1:1000 dilution)
https://www.genetex.com/Product/Detail/EIF6-antibody-N1C3-2/GTX117971

and anti-actin antibodies (Sigma, #A2066, 1:1000 dilution).
https://www.sigmaaldrich.com/GB/en/product/sigma/a2066

Anti-rabbit IgG HRP-linked antibody (Cell Signalling, #7074; 1:5000) was used as the secondary antibody.
https://www.cellsignal.com/products/secondary-antibodies/anti-rabbit-igg-hrp-linked-antibody/7074

Drosophila proteins were visualised using:

anti-Gapdh (Sigma #G9545, 1:20,000 dilution)
https://www.sigmaaldrich.com/GB/en/product/sigma/g9545

anti-eIF6 (GeneTex, #GTX117971, 1:1,000 dilution)
https://www.genetex.com/Product/Detail/EIF6-antibody-N1C3-2/GTX117971

anti-FLAG (Abcam, #Ab1257, 1:20,000 dilution)
https://www.citeab.com/antibodies/761220-ab1257-anti-ddddk-tag-binds-to-flag-tag-sequence-a

Secondary antibodies were all used at 1:10,000 dilution:
anti-mouse IgG, HRP-conjugated (Sigma-A5287)
https://www.sigmaaldrich.com/GB/en/product/sigma/a5278

anti-rabbit IgG, HRP-conjugated (Cell Signalling 7074)
https://www.cellsignal.com/products/secondary-antibodies/anti-rabbit-igg-hrp-linked-antibody/7074

anti-goat IgG, HRP conjugated (Santa Cruz, sc-2020) antibody.
https://datasheets.scbt.com/sc-2020.pdf

Validation

All used antibodies are standard and commonly used antibody clones, all validated by the companies (Biolegend or BD Biosciences) for their performance in flow cytometry, including specificity testing (1-3 target cell types) and brightness testing (incl. QC testing of serial dilutions).

Eukaryotic cell lines

Policy information about cell lines and Sex and Gender in Research

Cell line source(s)	HEK293T cell line was obtained from Sigma (12022001)
Authentication	PCR and genomic sequencing
Mycoplasma contamination	All cell lines were negative as inferred by negative PCR results
Commonly misidentified lines (See ICLAC register)	No commonly misidentified cell lines were used in this study

Animals and other research organisms

Policy information about studies involving animals; ARRIVE guidelines recommended for reporting animal research, and Sex and Gender in Research

Laboratory animals

We used Drosophila strains listed in Supplementary Tables 3 and 4 with sources included. Either adult flies or larvae were used as described in Methods section and Figure legends.

Drosophila Strains
Wild type (WT) w1118, J. Root (University of Cambridge UK)
SbdsP w1118; PBac{WH}CG8549f01686/TM6B, Tb1, Exelixis (Harvard)
da-GAL4 w*; P{GAL4-da.G32}UH1, Bloomington Drosophila Stock Centre
UAS-EIF6 w1118; pUAS-EIF6-FLAG, Alan J Warren lab
UAS-EIF6-I58T w1118; pUAS-EIF6-I58T-FLAG, Alan J Warren lab
UAS-EIF6-R96W w1118; pUAS-EIF6-R96W-FLAG, Alan J Warren lab
UAS-EIF6-N106S w1118; pUAS-EIF6-N106S-FLAG, Alan J Warren lab

Wild animals	The study did not include any wild animals.
Reporting on sex	Results are valid for both sexes
Field-collected samples	The study did not include any specimens, animals or samples collected from the field.
Ethics oversight	Ethics approval not required for experiments on invertebrates

Note that full information on the approval of the study protocol must also be provided in the manuscript.

Flow Cytometry

Plots

Confirm that:

☒ The axis labels state the marker and fluorochrome used (e.g. CD4-FITC).

☒ The axis scales are clearly visible. Include numbers along axes only for bottom left plot of group (a 'group' is an analysis of identical markers).

☒ All plots are contour plots with outliers or pseudocolor plots.

☒ A numerical value for number of cells or percentage (with statistics) is provided.

Methodology

Sample preparation	Samples were peripheral blood and/or bone marrow samples from individuals. Samples were processed as following: RBC lysis and isolation of mononuclear cells (MNCs) before staining with antibodies for flow cytometry.
Instrument	Phenotyping was done on FACSCanto (BD Biosciences). Flowsorting (2 samples) was done on FACSAria III or Influx (BD Biosciences).
Software	Data acquisition was performed using FACSDiva software (BD Biosciences) v8. Data analysis of phenotyping data was performed with FlowJo (version 10.6.1) software.
Cell population abundance	It was not possible to analyse post-sort fraction in the case of stem/progenitor cells due to low cell numbers. However, purity of flow-sorting was verified by sorting other cell fractions from the same sample and re-analysing these post-sort fractions.
Gating strategy	The FSC/SSC gate was set using the manual gating tool to exclude debris and small particles and to identify both the lymphocyte fraction and monocyte/granulocyte fraction of cells. The gating strategy after FSC/SSC gating was: gating for

single cells followed by live cells (7AAD-negative fraction), with subsequent exclusion of CD3/CD19 positive cells to exclude lymphocytes, followed by gating for CD34-positive progenitors. (Example in Supplementary Fig. 1).

☒ Tick this box to confirm that a figure exemplifying the gating strategy is provided in the Supplementary Information.
